# Ecofriendly Sunlight‐Mediated Nontoxic Bimetallic Nanoparticles: Synthesis, Reusable Catalytic Membrane, and Biosensor Applications

**DOI:** 10.1002/advs.202503120

**Published:** 2025-04-30

**Authors:** Samy M. Shaban, Sihyeok Kim, N. M. El Basiony, Jincymol Kappen, Mohamed H. Mostafa, Ahmed Y. Elbalaawy, Mohamed R. Elmasry, Jihoon Shin, Il Jeon, Dong‐Hwan Kim

**Affiliations:** ^1^ School of Chemical Engineering Sungkyunkwan University (SKKU) Suwon 16419 Republic of Korea; ^2^ Egyptian Petroleum Research Institute Nasr City Cairo 11727 Egypt; ^3^ Biomedical Institute for Convergence at SKKU (BICS) Sungkyunkwan University (SKKU) Suwon 16419 Republic of Korea; ^4^ Department of Nano Engineering Department of Nano Science and Technology SKKU Advanced Institute of Nanotechnology (SAINT) Sungkyunkwan University (SKKU) Suwon 16419 Republic of Korea; ^5^ SKKU Global Research Center (SGRC) Sungkyunkwan University (SKKU) Suwon 16419 Republic of Korea; ^6^ New Industry Creation Hatchery Center(NICHe) Tohoku University Sendai Sendai 980‐8576 Japan

**Keywords:** Ag–Cu bimetallic nanoparticles, Gemini surfactant, H₂O₂ detection, membrane‐assisted 4‐NP reduction, peroxidase‐mimic activity, sunlight‐induced reduction

## Abstract

Bimetallic nanoparticles (BMNPs) combine the desirable properties of two distinct metals that outperform conventional monometallic nanoparticles (NPs). This work presents a novel ecofriendly silver‐copper (Ag–Cu) BMNPs synthesis using sunlight as a green reducing agent, enableing rapid Ag–Cu BMNPs formation at room temperature within 10 min. This method exploiting the facile reduction of Ag⁺ to Ag⁰, which subsequently mediates the reduction of Cu^2^⁺ to Cu⁰ via water radiolysis‐generated species. The Ag–Cu BMNPs were integrated into two reusable catalytic membranes: PTFE@Ag–Cu, formed by immobilizing Ag–Cu BMNPs onto a polytetrafluoroethylene (PTFE) syringe filter, and ACF@Ag–Cu, synthesized via in‐situ growth of Ag–Cu BMNPs on activated carbon fiber (ACF) cloth. PTFE@Ag–Cu displays exceptional performance and reusability, converting 2100 mL of 0.15 mM *p*‐nitrophenol to *p*‐aminophenol over 105 cycles at a flow rate of 20 mL min^−1^. The Ag–Cu BMNPs also exhibit peroxidase‐mimic activity, enabling colorimetric H_2_O_2_ detection with a range of 0–200 mM and a limit of detection (LOD) of 13.3 µM in solution. Further, the Ag–Cu nanoenzyme demonstrates strong potential for electrochemical glucose detection, achieving an LOD of 0.1 µM and sensitivity of 5221 µA × 10^−6^ m⁻^1^ cm⁻^2^.

## Graphical abstract

A novel sunlight‐driven synthesis produces Ag–Cu bimetallic nanoparticles (NPs) in just 10 min using nontoxic surfactants. These high‐quality NPs show excellent catalytic activity, functioning as reusable polytetrafluoroethylene (PTFE) and activated carbon fiber (ACF) supported catalysts for efficient conversion of *p*‐nitrophenol to *p*‐aminophenol, and as enzymatic activators for sensitive colorimetric H₂O₂ detection and electrochemical glucose sensing.

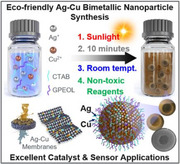

## 1 Introduction

Bimetallic nanoparticles (BMNPs) represent a fascinating frontier in nanoscience, combining two distinct metallic elements at the nanoscale to create alloys or core–shell configurations. The unique properties of BMNPs, arising from the intricate interactions between the constituent metals, result in enhanced catalytic activity, selectivity, and stability compared with their monometallic counterparts.^[^ ^1^, ^2^, ^3^ ^]^ This synergy has driven rapid growth in BMNP technology, with applications spanning biosensing, medical imaging, photothermal therapy, and catalysis.^[^ ^4^, ^5^, ^6^ ^]^ Well‐known BMNPs include Gold‐silver (Au–Ag),^[^ ^7^, ^8^ ^]^ Nikel‐gold (Ni–Au),^[^ ^9^ ^]^ silver‐palladium (Ag–Pd),^[^ ^10^ ^]^ and silver‐copper (Ag–Cu).^[^ ^11^ ^]^ Taking Ag–Cu as an example, although Ag nanoparticles (NPs) alone possess excellent antimicrobial properties, electrical conductivity, and catalytic capabilities, they are limited by durability issues and high costs. The incorporation of Cu addresses these challenges by preventing aggregation and enhancing stability; Cu serves as a stabilizer, improving both antimicrobial and catalytic properties while providing resistance to corrosion and oxidation.^[^ ^12^, ^13^, ^14^ ^]^ In addition, incorporating Cu reduces material costs. However, conventional BMNP synthesis methods, namely, coreduction,^[^ ^15^ ^]^ seed‐mediated growth,^[^ ^16^ ^]^ electrochemical deposition,^[^ ^17^ ^]^ and microemulsion,^[^ ^12^ ^]^ often rely on toxic reducing agents, high temperatures, and extended processing times. These methods pose environmental and safety concerns as well as have scalability and energy efficiency challenges. Moreover, recyclability issues often necessitate additional processes to recover a catalyst, potentially resulting in loss and toxicity.^[^ ^18^, ^19^, ^20^, ^21^, ^22^, ^23^, ^24^, ^25^, ^26^, ^27^ ^]^ For instance, Li et al.^[^ ^28^ ^]^ synthesized Ag–Cu BMNPs using toxic chemical reducing agents such as sodium citrate and NaBH_4_, requiring a long process of 24 h. Similarly, other methods employ toxic substances, such as hydrazinium chloride and hydrazine, often at elevated temperatures.^[^ ^15^, ^29^ ^]^ Darabdhara et al.^[^ ^30^ ^]^ synthesized Ag–Cu BMNPs via a multistep process involving heating at 200 °C. Some methods even necessitate calcination of Ag and Cu precursors at an extreme temperature of 600 °C.^[^ ^31^, ^32^, ^33^ ^]^ Given these challenges, there is a pressing need to develop ecofriendly, nontoxic, and facile methods for Ag–Cu BMNP synthesis. Ideally, such a methodology would be one‐step, high‐yield, and energy efficient, eliminating the need for toxic reducing agents. Moreover, conventional methods often struggle with recyclability, requiring additional processes to load a catalyst onto a magnetic substrate. This approach requires the use of external magnetic fields to collect catalysts from bulk solutions, potentially resulting in catalyst loss and toxicity issues.^[^ ^34^, ^35^, ^36^, ^37^, ^38^ ^]^

In this study, we propose a novel, ecofriendly method for synthesizing Ag–Cu BMNPs. The proposed method harnesses sunlight as a green reducing agent, enabling rapid transformation of Ag and Cu precursors into Ag–Cu NPs within 10 min at room temperature. This single‐pot, in situ photoreduction process eliminates the need for chemical reducing agents. Mechanistically, Ag⁺ is first reduced by sunlight to Ag^0^, which then indirectly reduces Cu^2+^ to Cu^0^ via activated species from water radiolysis. The chemical composition and physical morphology of the synthesized BMNPs were characterized using various analytical techniques. To further elucidate the BMNP synthesis mechanisms, Ag and Cu NPs were synthesized separately and compared with the BMNPs in terms of various perspectives. The synthesized ecofriendly sunlight‐mediated nontoxic Ag–Cu BMNPs exhibited exceptional catalytic properties compared to those of BMNPs synthesized using conventional methods (**Table** **1**).

**Table 1:** Comparison survey of bimetallic nanoparticles (BMNPs) syntheses reported to date.

Materials	Methods	Temperatures	Time	Toxicity	Year^[Ref.]^
Ag–Cu	Solar‐assisted reduction	R.T.	10 m	Non‐toxic	Our research
Cu–Ga	Coreduction	R.T	4 h >	Non‐toxic	2024^[^ ^39^ ^]^
Pd–Cu	Coreduction	60 °C	2 h >	Non‐toxic	2024^[^ ^40^ ^]^
Fe–Co	Coreduction	50 °C and 600 °C	24 and 3 h	Non‐toxic	2024^[^ ^41^ ^]^
Pt–Co	Coreduction	210 °C	40 m>	Toxic	2023^[^ ^42^ ^]^
Cu–Ni	Coreduction	R.T.	2 h	Toxic	2022^[^ ^43^ ^]^
Pt–Ag	Coreduction	70 °C	48 h	Non‐toxic	2022^[^ ^44^ ^]^
Ag–Cu	Coreduction	550 °C	4 h >	Non‐toxic	2022^[^ ^33^ ^]^
Ag–Cu	Coreduction	90 °C	1 h >	Toxic	2021^[^ ^15^ ^]^
Ni–Pt	Coreduction	100 °C	1 h	Toxic	2021^[^ ^45^ ^]^
Ag–Cu	Coreduction	R.T.	24 h	Toxic	2020^[^ ^28^ ^]^
Ag–Cu	Coreduction	600 °C	2 h	Non‐toxic	2019^[^ ^32^ ^]^
Ag–Cu	Co‐impregnation	500 °C	24 h >	Non‐toxic	2018^[^ ^31^ ^]^
Ni–Co	Coreduction	110 °C	1 h 45 min >	Toxic	2018^[^ ^46^ ^]^
Ag–Cu	Seed‐mediated growth	180 °C	4 h	Toxic	2017^[^ ^16^ ^]^
Ag–Cu	Coreduction	200 °C	2 h	Toxic	2016^[^ ^30^ ^]^
Ag–Cu	Electrochemical deposition	—	—	Toxic	2015^[^ ^17^ ^]^
Ag–Cu	Coreduction	R.T.	Not mentioned	Toxic	2015^[^ ^29^ ^]^
Pt–Co	Coreduction	600 °C	11 h >	Toxic	2014^[^ ^47^ ^]^

To demonstrate their application versatility, the synthesized BMNPs were employed as recyclable catalytic membranes, peroxidase‐mimic catalysts, and electrocatalytic sensors. For the recyclable catalyst application, two distinct systems were designed: a polytetrafluoroethylene (PTFE) based catalytic membrane filter for syringes and a catalytically activated carbon fiber (ACF). For the PTFE‐based catalytic membrane, the Ag–Cu BMNPs were filtered through to form a PTFE@Ag–Cu composite. For the ACF, the Ag–Cu BMNPs were grown directly in situ under sunlight, resulting in an ACF@Ag–Cu composite. The adhesion and uniformity of the BMNPs on both PTFE and ACF surfaces were confirmed using various observational techniques. The catalytic performance of the specialized membranes was evaluated by placing PTFE@Ag–Cu and ACF@Ag–Cu in a membrane holder, where they successfully catalyzed the conversion of *p*‐nitrophenol (*p*‐NP) to *p*‐aminophenol (*p*‐AP).

The Ag–Cu BMNPs demonstrated excellent catalytic activity for converting *p*‐NP to *p*‐AP, achieving a high apparent rate constant (*k*_app_) of 2.28 min⁻¹, completing the conversion within only 2 min. The PTFE@Ag–Cu and ACF@Ag–Cu catalytic membranes effectively removed toxic *p*‐NP, with the PTFE@Ag–Cu membrane showing superior performance; it successfully converted 2100 mL of *p*‐NP into *p*‐AP at a flow rate of 20 mL min^−1^ across 105 cycles without losing NPs or showing any decline in catalytic activity. Meanwhile, the ACF@Ag–Cu membrane converted 1500 mL at a flow rate of 10 mL min^−1^ over 75 cycles. Therefore, in terms of catalytic performance, the PTFE@Ag–Cu membrane is more efficient. However, the PTFE@Ag–Cu filter requires an additional process to immobilize Ag–Cu BMNPs. Meanwhile, the ACF@Ag–Cu membrane favors large‐scale production due to the capability for in situ growth over large areas and the ability to synthesize multiple membranes simultaneously. This highlights that both catalytic membranes have their respective strengths, and the choice between them should be based on the specific application requirements. Further, the enzymatic activity of Ag–Cu BMNPs in detecting H_2_O_2_ colorimetrically was examined by converting the o‐phenylene diamine (OPD) into orange‐yellow 2,3‐diaminophenazine (DAP). A remarkably wide range of 0–200 × 10^−3^ m and a low limit of detection (LOD) of 13.3 × 10^−6^ m were recorded. The Ag–Cu nanoenzyme was combined in agarose hydrogel to construct a colorimetric solid probe for H_2_O_2_ detection with 45.9 × 10^−6^ m LOD. In addition, the Ag–Cu BMNPs were tested as nanoenzymes in an electrochemical glucose detector. An LOD of 0.1 × 10^−6^ m and a sensitivity of 5221 µA × 10^−6^ m ^−1^ cm^−2^ were obtained, indicating the promising practical applicability for anti‐interferent biosensors.

The proposed method offers several advantages over conventional methods. It is ecofriendly and nontoxic, with rapid processing of 10 min at room temperature. The method yields high output while being energy efficient. Moreover, it provides excellent reusability without the need for magnetic substrates, addressing a significant limitation of conventional methods. The synthesized Ag–Cu BMNPs exhibited catalytic performance and glucose‐detecting capability comparable to those of BMNPs synthesized using conventional methods, with the added benefits of sustainability and cost effectiveness. This innovative approach represents a leap forward in the field of BMNP technology, offering a sustainable and efficient pathway for producing versatile materials.

## 2 Results and Discussion

### 2.1 Ecofriendly Synthesis of Ag–Cu BMNPs

**Figure 1:**
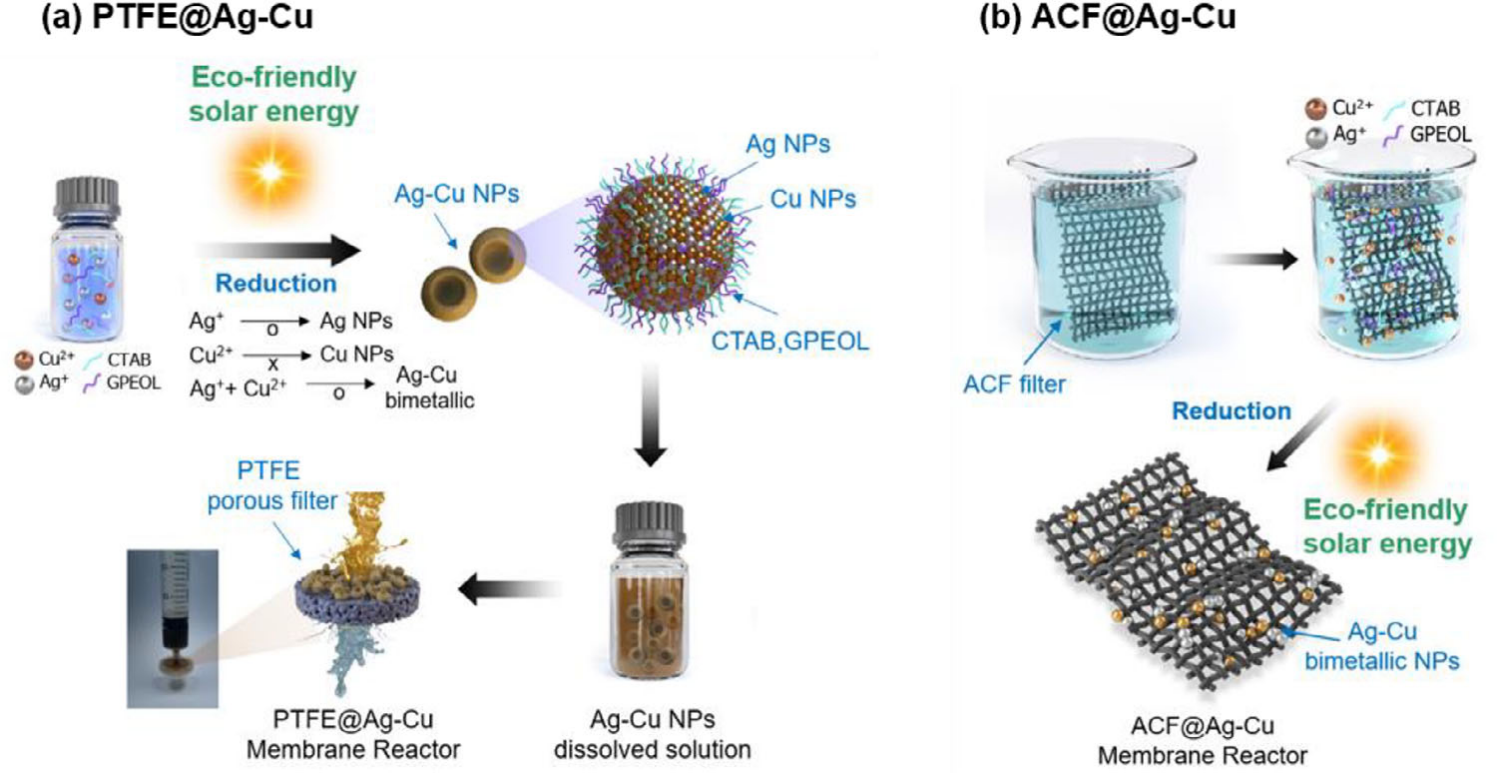
Schematic presentation of a) sunlight‐mediated synthesis of Ag–Cu bimetallic nanostructure and its immobilization on a syringe filter to construct PTFE@Ag–Cu catalytic membrane and b) sunlight‐mediated synthesis of ACF@Ag–Cu catalytic membrane for industrial waste treatments.

Ag–Cu BMNPs are commonly synthesized using methods such as coreduction, seed‐mediated growth, electrochemical deposition, microemulsion, and coprecipitation. However, these conventional methods often face challenges with efficiency, reproducibility, and structural control. Coreduction is the most widely used; however, it typically involves toxic reducing agents, high temperatures, complex processes, and high costs. To address these challenges, we proposed an ecofriendly, nontoxic, and energy efficient coreduction method: sunlight‐mediated, high‐throughput, one‐step coreduction synthesis (**Figure** **1**a). In this synthesis, AgNO₃ and Cu(NO₃)₂ were added to a mixture of hexadecyltrimethylammonium bromide (CTAB) and Gemini nonionic surfactant based on polyethylene oxide 1500 (GPEOL) solutions and exposed to sunlight for 10 min. The detailed role of surfactants are explained in Figure S1 (Supporting Information). The observed color change from aqua blue to brownish green confirmed the successful synthesis of Ag–Cu BMNPs (Figure S2, Supporting Information). Meanwhile, Ag NPs would appear brownish red, and CuO–Cu^2^⁺ would show no apparent color change. Unlike conventional methods, this process is free of toxic reducing agents, takes 10 min, and occurs at room temperature.

To evaluate the reusability of the Ag–Cu BMNPs, particularly for industrial wastewater treatment, we employed two strategies: filtering the BMNPs through a PTFE syringe filter (Figure 1a) and growing Ag–Cu BMNPs in situ on an ACF mesh surface (Figure 1b). Both the Ag–Cu NPs‐attached syringe filter and ACF mesh demonstrated excellent reusability, with no noticeable decline in catalytic performance, even after extended use. This is crucial for industrial applications, as conventional recycling methods often involve complex processing, such as magnetic separation, to retrieve NPs. For the Ag–Cu NPs‐attached syringe filter, 1‐mL distilled water was injected through a PTFE filter with a pore size of 0.1 µm, followed by injecting varying amounts of Ag–Cu BMNPs (0.25, 0.5, and 1 mL). The syringe filter was then rinsed with distilled water to remove excess unattached particles and dried at room temperature for 3 h (Figure 1a). For the ACF‐grown Ag–Cu BMNPs, we synthesized the NPs directly on ACF cloth under sunlight, which served as a green reducing agent (Figure 1b). A 5 cm × 5 cm piece of ACF cloth was first immersed in a mixed solution of 25‐mL CTAB (2 × 10^−3^ m) and 25‐mL GPEOL (0.5 × 10^−3^ m) for 5 min. Subsequently, 50‐mL AgNO₃ (50 × 10^−3^ m) and 50‐mL Cu(NO₃)₂ (200 × 10^−3^ m) were added, and the mixture was stirred for an additional 5 min. The impregnated ACF cloth was then exposed to sunlight for 20 min, resulting in a uniform decoration of Ag–Cu BMNPs on the ACF surface. Both approaches enable the large‐scale and mass production of Ag–Cu BMNPs, demonstrating their potential for industrial applications. The proposed method not only addresses the limitations of conventional synthesis methods but also supports sustainable and ecofriendly synthesis of BMNPs suitable for various catalytic applications.

### 2.2 Verification of Synthesized Ag–Cu BMNPs

**Figure 2:**
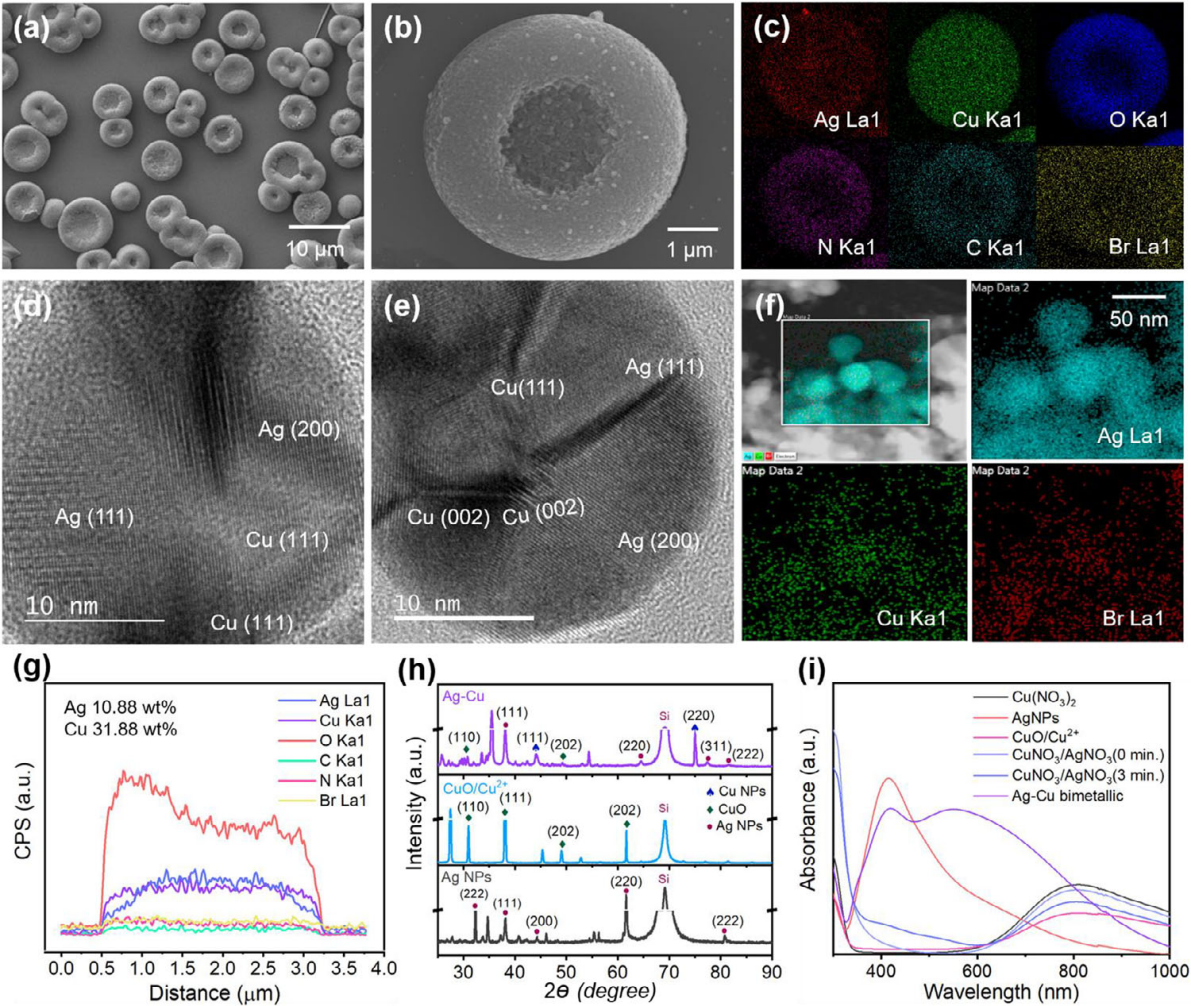
Scanning electron microscopy (SEM) images of a,b) Ag–Cu bimetallic nanoparticles (BMNPs) with different magnifications. c) Energy‐dispersive X‐ray (EDX) mapping of the Ag–Cu bimetallic structure with Ag, Cu, O, N, C, and Br elements. d,e) Transmission electron microscopy (TEM) images of Ag–Cu BMNPs. f) TEM–EDX mapping of Ag–Cu bimetallic structure with Ag, Cu, and Br elements. g) Composition of Ag–Cu BMNPs. h) X‐ray diffraction (XRD) spectra of Ag NPs, CuO–Cu^2^⁺, and Ag–Cu BMNPs. i) UV–Vis spectra of the Ag NPs, CuO–Cu^2^⁺, and Ag–Cu BMNPs under different preparation conditions.

Various analyses were performed to confirm the successful synthesis of Ag–Cu BMNPs using the proposed method. Scanning electron microscopy (SEM) images reveal the distinct doughnut‐like clusters of Ag–Cu BMNPs (**Figure** **2**a,b), which differ from the structures formed by Ag and CuO NPs individually (Figure S3a,b, Supporting Information). This unique microsized domain‐based morphology consists of closely aligned bimetallic NPs arranged without aggregation (Figure S4, Supporting Information). Although these NPs agglomerate to form larger microstructures, it is important to clarify that this agglomeration does not equate to aggregation. Instead, it facilitates the formation of highly porous domains, which significantly enhance the adsorption capacity and contribute to the superior catalytic activity of the bimetallic system. Energy‐dispersive X‐ray (EDX) mapping (Figure 2c) confirms the presence of Ag, Cu, O, N, C, and Br within the same microsized domain, highlighting the uniform distribution of Ag and Cu nanocrystals throughout the composite. The presence of O, N, C, and Br from the surfactant mixture reflects critical role of surfactants in assembling Ag and Cu into the unique structure without aggregation. Transmission electron microscopy (TEM) images of the Ag–Cu BMNPs (Figure 2d,e) show distinct lattice planes for Cu [111], [002] and Ag [111], [200], with corresponding *d*‐spacing values of 0.231, 0.248, 0.235, and 0.204 nm, confirming the Ag–Cu alloy formation, compared with the individual TEM data for Ag and CuO NPs (Figure S3c,d, Supporting Information). TEM–EDX mapping (Figure 2f) verifies the chemical composition of the NPs as Ag and Cu, with a weight ratio of approximately 1:3 (Figure 2g).

Spectral analyses further corroborate the chemical composition of the Ag–Cu BMNPs. X‐ray diffraction (XRD) patterns of Ag NPs, CuO–Cu^2^⁺, and Ag–Cu BMNPs (Figure 2h) confirm the presence of distinct crystal planes. The Ag NPs exhibit peaks at 2*θ* values of 32.4°, 38.14°, 44.4°, 62.5°, and 81.6°, corresponding to the (222), (111), (200), (220), and (222) planes of face‐centered cubic (fcc) Ag, respectively, as per JCPDS 04–0783.^[^ ^40^, ^41^ ^]^ The CuO–Cu^2^⁺ spectrum shows peaks at 2*θ* values of 31.2°, 38.08°, 49.06°, and 61.7°, indexed to the (110), (111), (202), and (202) planes of monoclinic CuO, respectively (JCPDS No. 01‐080‐0076 and JCPDS 5–0661).^[^ ^48^, ^49^, ^50^ ^]^ The Ag–Cu BMNPs exhibit peaks characteristic of fcc Ag at 2*θ* values of 38.36°, 64.4°, 77.6°, and 82.04° ((111), (220), (311), and (222) planes, respectively and additional peaks at 43.7° and 75.32°, indicated the presence of (111) and (220) planes confirming the presence of metallic Cu (JCPDS 04–0836) along with CuO.^[^ ^50^, ^51^, ^52^ ^]^ These results align with TEM findings, validating the successful synthesis of Ag–Cu BMNPs.

Ultraviolet–visible (UV–Vis) absorption spectra reveal two distinct extinction peaks at *λ* = 426 and 550 nm, corresponding to Ag and Cu NPs within the Ag–Cu structure, respectively.^[^ ^5^ ^]^ Ag NPs alone displayed a surface plasmon resonance peak at *λ* = 415 nm, showing a blue shift of 11 nm compared with Ag–Cu BMNPs (Figure 2i). The UV–Vis spectrum of Cu(NO₃)₂ alone showed a broad peak around 600 nm with reduced intensity at 785 nm, indicating the challenge of synthesizing Cu NPs independently. When Ag and Cu precursors were combined, a rapid color change occurred within 10 min, indicating the formation of the Ag–Cu bimetallic structure. The UV–Vis spectra of the AgNO₃ and Cu(NO₃)₂ mixture without sunlight exposure showed no peaks between 400 and 700 nm. However, after 3 min of sunlight exposure, two small peaks appeared (light blue curve), corresponding to the initial growth of Ag and Cu NPs and the associated color change. After 10 min, the Ag–Cu bimetallic structure was fully formed, evidenced by two significant surface plasmon resonance peaks at *λ* = 426 and 550 nm for Ag and Cu, respectively (dark blue curve). These peaks were recorded after diluting the sample 10‐fold, confirming the successful formation of the Ag–Cu bimetallic structure.

### 2.3 Investigation of Ag–Cu BMNP Synthesis Mechanism

**Figure 3:**
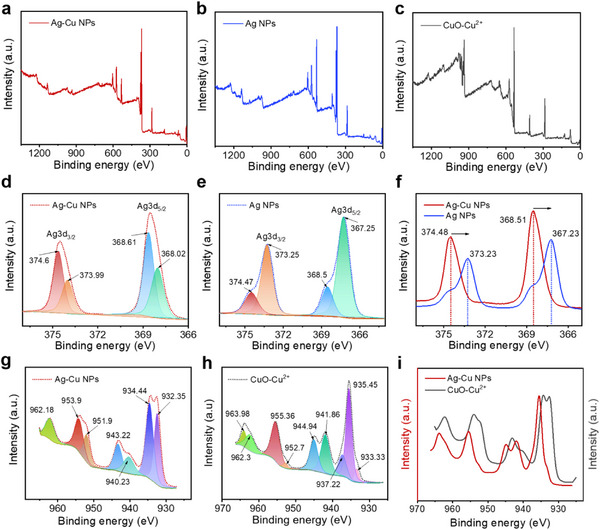
(a‐c) X‐ray photoelectron spectroscopy (XPS) high spectral survey of (a) Ag–Cu bimetallic nanoparticles (BMNPs), (b) Ag nanoparticles (NPs), and (c) CuO–Cu^2^⁺. The high‐resolution Ag 3d X‐ray photoelectron spectroscopy (XPS) of d) Ag–Cu and e) Ag NPs and f) Ag 3d spectrum overlapping between Ag–Cu and Ag NPs demonstrating a shift in binding energy. The high‐resolution Cu 2p XPS of g) Ag–Cu BMNP and h) CuO–Cu^2^⁺ and i) Cu 2p spectrum overlapping between the Ag–Cu BMNP and CuO–Cu^2^⁺ demonstrating a shift in binding energy.

To better understand the valence band structure and chemical composition of the synthesized Ag–Cu BMNPs, X‐ray photoelectron spectroscopy (XPS) analysis was performed and compared with XPS data obtained from Ag NPs and CuO–Cu^2^⁺. **Figure** **3**a–c shows the XPS survey spectra of Ag–Cu BMNPs, Ag NPs, and CuO–Cu^2^⁺, respectively. In the Ag–Cu BMNPs (Figure 3a), characteristic peaks corresponding to the Cu 2p orbitals confirm the successful incorporation of Cu into the composite. The high‐resolution XPS spectra (Figure 3d) display the Ag 3d₃/₂ and Ag 3d₅/₂ orbitals in the Ag–Cu composite at 374.48 and 368.53 eV, respectively, with a 6.0 eV separation, similar to the Ag 3d peaks observed in the Ag NPs (Figure 3e: 373.7 and 367.6 eV). These results indicate the presence of metallic Ag (Ag⁰) in the Ag–Cu and Ag NP samples. The slight shift in the binding energies of Ag 3d₃/₂ and Ag 3d₅/₂ orbitals in the Ag–Cu BMNPs compared with Ag NPs (Figure 3f) is attributed to electronic structural changes in Ag due to the introduction of Cu, which induces shifts in the XPS peak positions.^[^ ^3^, ^53^ ^]^ Figure 3g shows peaks at 951.9 and 932.35 eV with a 20 eV splitting, corresponding to Cu⁺/Cu⁰ states in the Ag–Cu composite.^[^ ^3^, ^54^, ^55^, ^56^ ^]^ Additional peaks at 934.44 and 953.99 eV, along with satellite peaks at 940.23, 943.22, and 962.1 eV, indicate the presence of Cu^2^⁺ due to surface oxidation of Cu atoms when the Ag–Cu composite is exposed to air.^[^ ^3^, ^57^ ^]^ Figure 3h shows the XPS spectrum of the synthesized CuO–Cu^2^⁺ catalyst, with peaks at 935.45, 937.22, and 955.36 eV, along with satellite peaks at 941.86, 944.94, 963.3, and 963.98 eV, confirming the presence of Cu^2^⁺^[^ ^58^, ^59^ ^]^ and a minor fraction of Cu⁺/Cu⁰ at 933.33 and 952.66 eV. Meanwhile, the Cu⁺/Cu⁰ peak ratio (932.23 eV) to Cu^2^⁺ peak (934.44 eV) in the Ag–Cu BMNPs is 0.94, whereas the CuO–Cu^2^⁺ prepared without AgNO₃ shows a much lower ratio of 0.1 for the peaks at 933.33 to 935.45 eV. This lower ratio reflects the difficulty of synthesizing Cu NPs under the same conditions as Ag or Ag–Cu NP synthesis, consistent with the UV–Vis results shown in Figure 2i. The observed shift in the XPS spectra (Figure 3i) is attributed to the formation of a Cu–Ag alloy. The interaction between the two metals results in a change in the electronic structure, leading to a shift in the binding energy of the core levels.^[^ ^3^, ^53^ ^]^ This phenomenon is well‐documented in the literature and has been observed in other bimetallic systems.

To delve deeper into the mechanism of Ag–Cu BMNPs, through the photoreduction of Ag to induce the formation of metallic Cu under mild and ecofriendly conditions, different control experiments were conducted with/without AgNO_3_ and the mixed CTAB/GPEOL solution as well as with individual surfactants. In summary, the use of a binary surfactant system (CTAB and GPEOL) along with AgNO₃ and Cu(NO₃)₂ resulted in the successful formation of Ag–Cu BMNPs within 10 min of sunlight exposure at room temperature. In contrast, when only a single surfactant (either CTAB or GPEOL) was used, the reaction proceeded significantly slower, yielding minimal product even after 24 h (Video S1 and Figure S5a, Supporting Information). This highlights the synergistic effect of the binary surfactant system in accelerating the reaction and enhancing nanoparticle formation.^[^ ^60^, ^61^, ^62^ ^]^ The presence of AgNO_3_ is essential for the formation of metallic Cu, as confirmed by separate experiments with AgNO_3_ and Cu(NO_3_)_2_ alone under the same conditions. Ag NPs were formed within 10 min in the absence of Cu(NO_3_)_2_ (Figure S2, Supporting Information), whereas Cu(NO_3_)_2_ alone did not show any color change even after a week of exposure to sunlight (Cu NPs were not detected by UV, Figure 2i). These individual experiments highlight the potential importance of AgNO_3_ in inducing the Cu(NO_3_)_2_ reduction to metallic Cu^0^ to construct the Ag–Cu BMNPs.

To understand why AgNO_3_ plays a pivotal role in reducing the Cu⁺^2^ to Cu^0^, we first need to understand how the individual Ag NPs are formed in the absence of the Cu precursor under the same experimental conditions. Ag NPs are prepared through a photoreduction mechanism using sunlight as a surplus green reducing agent, with a CTAB/GPEOL surfactant mixture acting as a capping and coreducing agent. When the AgNO_3_/surfactant mixture was exposed to sunlight, a rapid color change that stabilized after 10 min was observed, indicating the formation of Ag NPs (Figures S2 and S3a, Supporting Information). However, when we conducted a control experiment with either the AgNO_3_/surfactant mixture in the absence of sunlight or AgNO_3_ in the presence of sunlight without CTAB/GPEOL, we did not observe any indication of Ag NP formation, even after one month, clarifying the relevance of both sunlight and the CTAB/GPEOL mixture for Ag NP formation. Based on this, we conclude that the reaction mechanism relies on the sunlight promoting the water radiolysis that generates some active species, such as OH^•^, H_2_O_2_, H_2_, and H^•^, as well as solvated electrons.^[^ ^63^, ^64^, ^65^, ^66^ ^]^ The H_2_O_2_, H_2_, and solvated electrons can act as reducing agents; although their concentrations are insufficient to induce Ag⁺ reduction into Ag NPs in the absence of the surfactant mixture, even after weeks of exposure to sunlight. However, when the CTAB/GPEOL mixture was present, the released H^•^ and OH^•^ from water radiolysis could be scavenged CTAB and GPEOL to produce H_2_O_2_ and H_2_, thereby considerably enhancing the amount of the liberated H_2_O_2_, H_2_, and solvated electrons in accordance to the Le Chatelier's principle.^[^ ^67^ ^]^ The interaction of these radicals with surfactant molecules may convert them into radical analogues (e.g., CTAB• and GPEOL•), with probability of aggregation or polymerization,^[^ ^68^, ^69^ ^]^ altering their electronic properties enhancing π–π or n–π transitions and contributing to the substantial absorbance increase from approximately 3.5 a.u. (without dilution) to 1.61 a.u. (after 10× dilution due to instrument limitations), as shown in Figure S5b (Supporting Information). Therefore, sunlight initiates the production of OH^•^, H_2_O_2_, H_2_, and H^•^ as well as solvated electrons whereas the surfactant enhances the concentrations of H_2_O_2_, H_2_, and solvated electrons, resulting in a rapid reduction of Ag⁺ to Ag NPs.^[^ ^70^ ^]^

According to the standard electrode potential (*E*_0_) of the pairs Ag^+^/Ag^0^ (+0.8 V) and Cu^2+^/Cu^0^ (+0.34 V),^[^ ^71^ ^]^ Ag^⁺^ is easier and faster to reduce than Cu^2+^.^[^ ^72^ ^]^ Therefore, in the presence of sunlight and the CTAB/GPEOL mixture, during the Ag–Cu BMNP synthesis, Ag^+^ will be reduced into Ag^0^ first. This is followed by the release of H_2_O_2_, H_2_, and solvated electrons in large quantities, which promote the reduction of Cu^2+^ to Cu^0^, leading to the Ag–Cu bimetallic structure formation. As shown in Figure 2i, both surface plasmonic resonance peaks for Ag and Cu NPs were observed after 3 min of exposing AgNO_3_/Cu(NO_3_)_2_/CTAB/GPEOL to sunlight and continued to increase until reaching maximum intensity after 10 min of sunlight exposure (Figure 2i, dark blue curve), indicating the Ag–Cu bimetallic structure formation. The ease of reduction of Ag⁺ to Ag^0^ is exploited for the indirect reduction of Cu^2^ to Cu^0^ via the released H_2_O_2_, H_2_, and solvated electron species from Ag reduction. In addition, the overflow of the released H_2_ can induce pores in the synthesized Ag–Cu BMNPs.^[^ ^3^ ^]^ Meanwhile, the Cu(NO_3_)_2_ solution alone without AgNO_3_ did not show any color change, even after one week of exposure to sunlight (Cu NPs were not detected by UV; Figure 2i), although XPS revealed a small ratio of Cu^0^ to Cu^2^⁺ (Figure 3g). These individual experiments emphasize the relevance of AgNO_3_ in the reduction of Cu(NO_3_)_2_ to metallic Cu^0^, thereby forming Ag–Cu BMNPs.

### 2.4 Applications of Synthesized Ag–Cu NPs

Having confirmed the chemical composition, molecular structure, and synthesis mechanism of the Ag–Cu BMNPs, we investigated their catalytic applications. One key application is the conversion of *p*‐NP, a toxic pollutant commonly found in industrial wastewater, to *p*‐AP, a less harmful compound widely used in the pharmaceutical and dye industries. This conversion not only demonstrates the catalytic activity of Ag–Cu BMNPs but also showcases an environmentally beneficial process that converts a hazardous substance into a more valuable and safer product.

The conversion of *p*‐NP to *p*‐AP in the presence of NaBH₄^[^ ^73^ ^]^ serves as an indicator of catalytic performance and highlights the practical environmental benefits of using the catalyst. Upon the addition of NaBH₄, the absorption peak of *p*‐NP shifts from *λ* = 314 to 400 nm due to the formation of a resonating nitrophenolate intermediate. Without the catalyst, *p*‐NP shows negligible transformation over 12 h, even with NaBH₄, as evidenced by the constant peak intensity at *λ* = 400 nm. To compare the performance of the Ag–Cu bimetallic catalyst with monometallic catalysts, different concentrations of Ag NPs, CuO–Cu^2^⁺, and Ag–Cu BMNPs were tested against 0.1 × 10^−3^ and 0.15 × 10^−3^ m *p*‐NP dyes. Although both Ag NPs and CuO–Cu^2^⁺ exhibited adequate catalytic activity, the Ag–Cu bimetallic catalyst exhibited notably enhanced performance, demonstrating the benefits of doping between Ag and Cu compared with the monometallic nanostructures (Figures S6–S9, Supporting Information). As shown in Figure S6 (Supporting Information), both monometallic and bimetallic catalysts successfully converted toxic 0.1 × 10^−3^ m *p*‐NP to nontoxic *p*‐AP, indicated by the decreasing peak intensity of *p*‐NP at *λ* = 400 nm and the emergence of a new peak at *λ* = 300 nm for p‐AP over time. Using 6‐µL Ag–Cu BMNPs, the complete conversion of 0.1 × 10^−3^ m *p*‐NP to p‐AP was achieved within 2 min (Figure S6a, Supporting Information), whereas the same concentration of Ag NPs and CuO–Cu^2^⁺ required 12 and 17 min, respectively, for the complete conversion. Increasing the dye concentration to 0.15 × 10^−3^ m slightly reduced catalytic activity. The slight decrease in the catalytic activity at higher *p*‐NP concentrations is due to dye saturation on the catalyst surface, where excess *p*‐NP may lead to competitive adsorption at active sites, reducing the turnover frequency by limiting efficient electron transfer from BH₄⁻ to *p*‐NP. At high *p*‐NP concentrations, diffusion limitations can occur, where the substrate movement toward active catalytic sites becomes a limiting factor, and increased substrate layers may hinder access to active sites.^[^ ^74^, ^75^ ^]^ These limitations may result in concentration gradients, further reducing the catalysis rate. Ag–Cu BMNPs (6 µL) converted *p*‐NP to *p*‐AP in 5 min, whereas Ag NPs and CuO–Cu^2^⁺ required 14 and 23 min, respectively (Figures S7–S9, Supporting Information). These results confirm that, compared with monometallic catalysts, the synergistic doping of Cu and Ag in the bimetallic structure improves *p*‐NP conversion. Increasing the catalyst dosage further reduced the time required for *p*‐NP conversion (Figures S6–S9, Supporting Information). The dye removal efficiency was calculated as follows:

(1) $$\mathrm{Removal}\;\mathrm{of}\;\mathrm{dye}\;=\left(\frac{C_{o}-C_{t}}{C_{o}}\right)\times 100\%$$

where *C*_o_ and *C*_t_ denote the initial and final *p*‐NP concentrations, respectively. The efficiency of *p*‐NP removal increased with higher catalyst dosages, facilitating faster *p*‐NP conversion to *p*‐AP, attributable to the increased adsorption surface area.^[^ ^76^, ^77^ ^]^ Specifically, using 1, 2, 4, and 6 µL of Ag–Cu BMNPs achieved *p*‐NP removal efficiencies of 95.17%, 96.23%, 97.82%, and 99.9% within 16, 9, 3.5, and 2 min, respectively, when removing 0.1 × 10^−3^ m *p*‐NP (Figure S10, Supporting Information). These results demonstrate the superior catalytic performance of the Ag–Cu BMNPs, demonstrating their potential for practical environmental applications by efficiently converting toxic pollutants into safer, invaluable products.

The kinetic rate constant (*k*_app_) clarified that the reduction of *p*‐NP followed a pseudo‐first‐order model (Figure S11, Supporting Information), showing dose‐dependent catalytic activity. Ag–Cu BMNPs outperformed monometallic catalysts, with *k*_app_values of 2.28, 0.3045, and 0.1902 min⁻¹ for Ag–Cu BMNPs, Ag NPs, and CuO–Cu^2^⁺, respectively (Table S1, Supporting Information). The superior catalytic performance of Ag–Cu BMNPs is attributed to an increased number of active sites, maximizing the total surface area. Further details, including comparative studies, are provided in Tables S1 and S2 (Supporting file).

#### 2.4.1 Ag–Cu NPs as Reusable Catalytic Membranes

**Figure 4:**
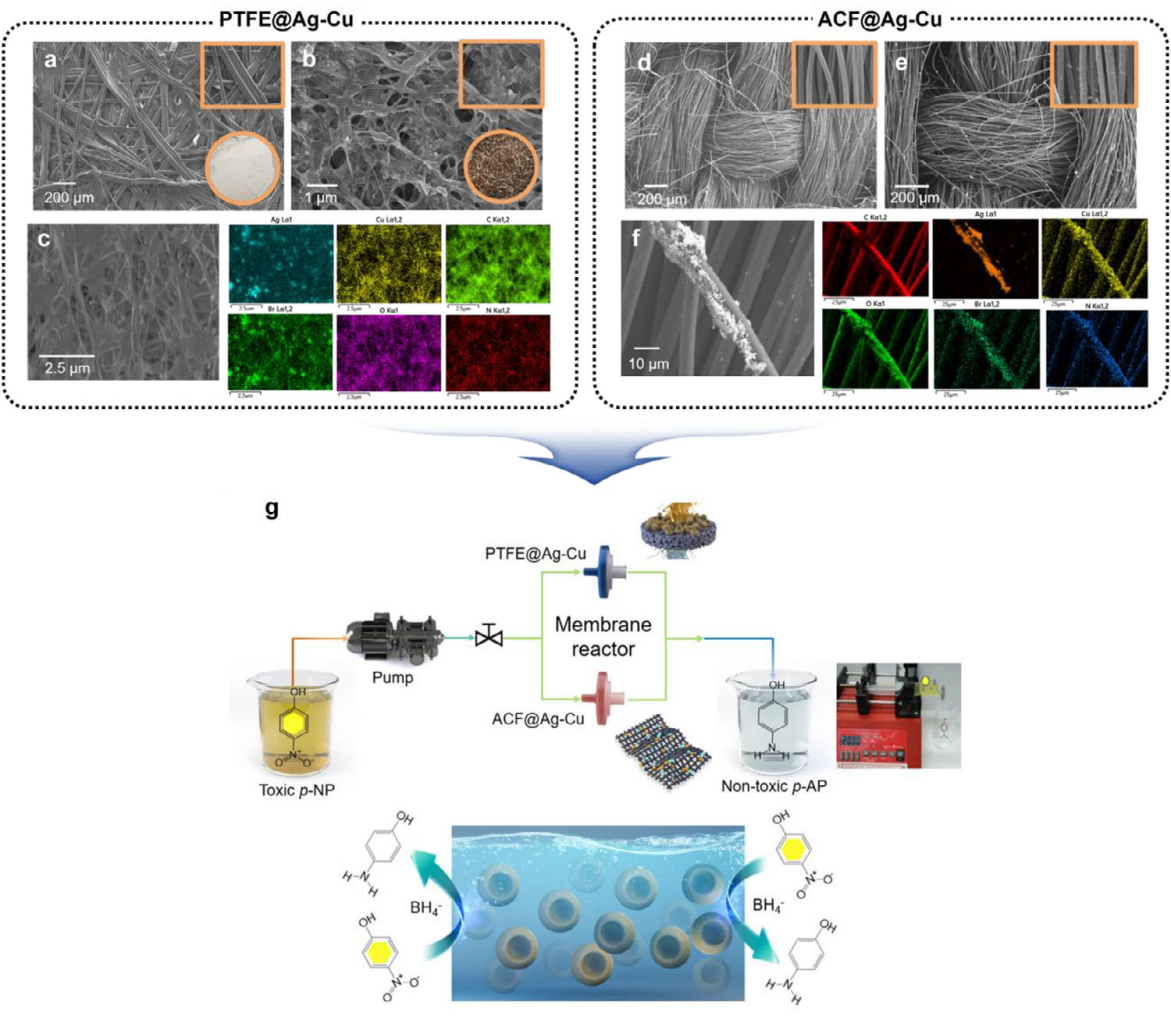
Scanning electron microscopy (SEM) images of a) blank polytetrafluoroethylene (PTFE) filter membrane with 0.1 × 10^−6^ m pore size without immobilization of nanoparticles (NPs) (inset: high‐magnification SEM and real photo), b) PTFE filter membrane with 0.1 × 10^−6^ m pore size after immobilization with Ag–Cu bimetallic nanoparticles (BMNPs) (inset: high‐magnification SEM and real photo), c) energy‐dispersive X‐ray (EDX) mapping of PTFE@Ag–Cu with Ag, Cu, O, N, C, and Br elements; SEM images of d) blank activated carbon fiber (ACF) fiber without NPs (inset: high‐magnification SEM) and e) ACF after direct in situ growth of Ag–Cu bimetallic (inset: high‐magnification SEM). f) EDX mapping of ACF@Ag–Cu with Ag, Cu, O, N, C, and Br elements. g) Schematic presentation and real image describing how the fabricated PTFE@Ag–Cu and ACF@Ag–Cu catalytic membranes are used for pollutant treatment.

To fully leverage the catalytic performance of Ag–Cu BMNPs, address catalyst recovery issues, and enhance their industrial applicability, we employed two strategies: embedding Ag–Cu BMNPs directly into a syringe filter (PTFE@Ag–Cu) and in situ growth of Ag–Cu BMNPs on an ACF mesh (ACF@Ag–Cu). SEM images of the PTFE filter before (**Figure** **4**a) and after (Figure 4b) filtering Ag–Cu BMNPs confirm the thorough coating of the filter fibers with bimetallic catalysts, whereas SEM–EDX images verify the chemical composition of the NPs (Figure 4c). For the ACF mesh, SEM images also confirm the successful in situ growth of Ag–Cu BMNPs (Figure 4d–e). The ACF@Ag–Cu nanostructure was engineered via in situ growth using sunlight as a green reducing agent. The ACF@Ag–Cu membrane effectively eliminates toxic materials without requiring catalyst recovery. The ACF substrate offers several benefits, including high porosity, excellent packing density, and rapid adsorption capabilities, making it ideal for catalytic applications.^[^ ^78^, ^79^ ^]^ EDX mapping shows the distributions of Ag, Cu, and surfactants on the ACF (Figure 4f), indicating strong binding between the ACF and metal precursors facilitated by functional groups in the surfactants.

To test the PTFE@Ag–Cu membrane, a 20‐mL solution of 0.15 × 10^−3^ m *p*‐NP and NaBH_4_ was injected through a syringe pump at 20 mL min^−1^ (Figure 4g). The catalytic membrane efficiently converted *p*‐NP to *p*‐AP, achieving complete conversion in just 1 min (Video S2, Supporting Information). Catalytic membranes of 0.25, 0.5, and 1 mL were reusable for 40, 75, and 105 cycles, respectively, converting 800, 1500, and 2100 mL of *p*‐NP with nearly consistent efficiency (Figure S12, Supporting Information). SEM analysis after 105 cycles showed that the Ag‐Cu BMNPs remained intact on PTFE fibers without significant loss (Figure S13, Supporting Information). Similarly, the ACF@Ag–Cu membrane was tested by inserting a 25‐mm ACF@Ag–Cu disc into a syringe filter. A 10‐mL solution of 0.15 × 10^−3^ m *p*‐NP and NaBH_4_ was pumped at 10 mL min^−1^ (Figure 4g), achieving a complete conversion of 20‐mL *p*‐NP in 2 min (Video S3, Supporting Information). The ACF@Ag‐Cu membrane was reusable for 75 cycles at the same rate, converting 1500‐mL *p*‐NP with identical conversion efficiency (Figure S12d, Supporting Information).

The toxicity of Ag NPs, CuO–Cu^2^⁺, and Ag–Cu BMNPs was evaluated using an MTT assay (Figure S14a, Supporting Information). Ag NPs and Ag–Cu BMNPs exhibited higher cell viability at concentrations of 0.1 and 0.2 µL mL^−1^, respectively, compared with CuO–Cu^2^⁺, which showed low viability. Additionally, distilled water was passed through the PTFE@Ag–Cu and ACF@Ag–Cu membranes to assess the potential detachment of the catalysts. The cell viability of the drained water was nearly 100%, confirming the effective immobilization of Ag–Cu BMNPs on both membranes (Figure S14b,c, Supporting Information).

The prepared catalysts were employed to reduce *p*‐NP to *p*‐AP in the presence of NaBH_4_. The Ag–Cu bimetallic catalyst plays a crucial role in facilitating electron transfer from BH_4_⁻ to *p*‐NP, overcoming kinetic limitations despite the thermodynamically favorable potential difference. The proposed mechanism can be summarized in three primary stages. (1) Catalytic electron relay: Ag–Cu BMNPs act as an electron relay system, accepting electrons from BH₄⁻ and transferring them to the adsorbed *p*‐NP. Ag and Cu create a bimetallic interface that enhances electron mobility compared with monometallic catalysts, which lowers the activation energy of the reduction and accelerates reaction kinetics. Although thermodynamically favorable (*E*° of *p*‐NP/*p*‐AP = −0.76 V versus RHE; *E*° of H₃BO₃/BH₄⁻ = −1.33 V versus RHE),^[^ ^80^ ^]^ the reaction cannot proceed kinetically without a catalyst due to the significant potential difference between the *p*‐NP acceptor and BH₄⁻ donor.^[^ ^81^ ^]^ The reduction occurs as BH₄⁻ donates electrons and H₂ to *p*‐NP. (2) Intermediate formation and stabilization: In the reaction pathway, NaBH₄ initially donates electrons and protons, producing surface‐adsorbed hydrogen (H_ads_) intermediates on the Ag–Cu bimetallic catalyst. These intermediates subsequently react with *p*‐NP adsorbed on the catalyst surface to form a nitroso (–NO) intermediate, which further reduces to form *p*‐AP. This stepwise reduction is facilitated by the Ag–Cu surface, which not only stabilizes the intermediate species but also minimizes activation energy barriers.^[^ ^82^ ^]^ (3) Surface interactions: The surfactants (GPEOL and CTAB) stabilize the Ag–Cu BMNPs, enhancing their colloidal stability and providing functional groups that promote electrostatic and hydrogen‐bonding interactions with *p*‐NP. The surfactants provide multiple functional groups (carbonyl, double bond, ethylene oxides, and quaternary ammonium) that promote electrostatic and hydrogen‐bonding interactions between the *p*‐NP dye and catalyst, facilitating electron transfer from BH_4_⁻ to *p*‐NP.^[^ ^83^ ^]^ This increased adsorption aids in efficient electron transfer to p‐NP, whereas Ag–Cu BMNPs act as a platform for improved interaction and stabilization of intermediates. As shown in Figure 4g, adsorbed BH_4_⁻ donates electrons and H_2_ to *p*‐NP, facilitated by the active species on the Ag–Cu catalyst. These findings highlight the excellent catalytic potential of Ag–Cu BMNPs as reusable membranes for industrial applications.

#### 2.4.2 Ag–Cu NPs as Peroxidase‐Mimic Activator

**Figure 5:**
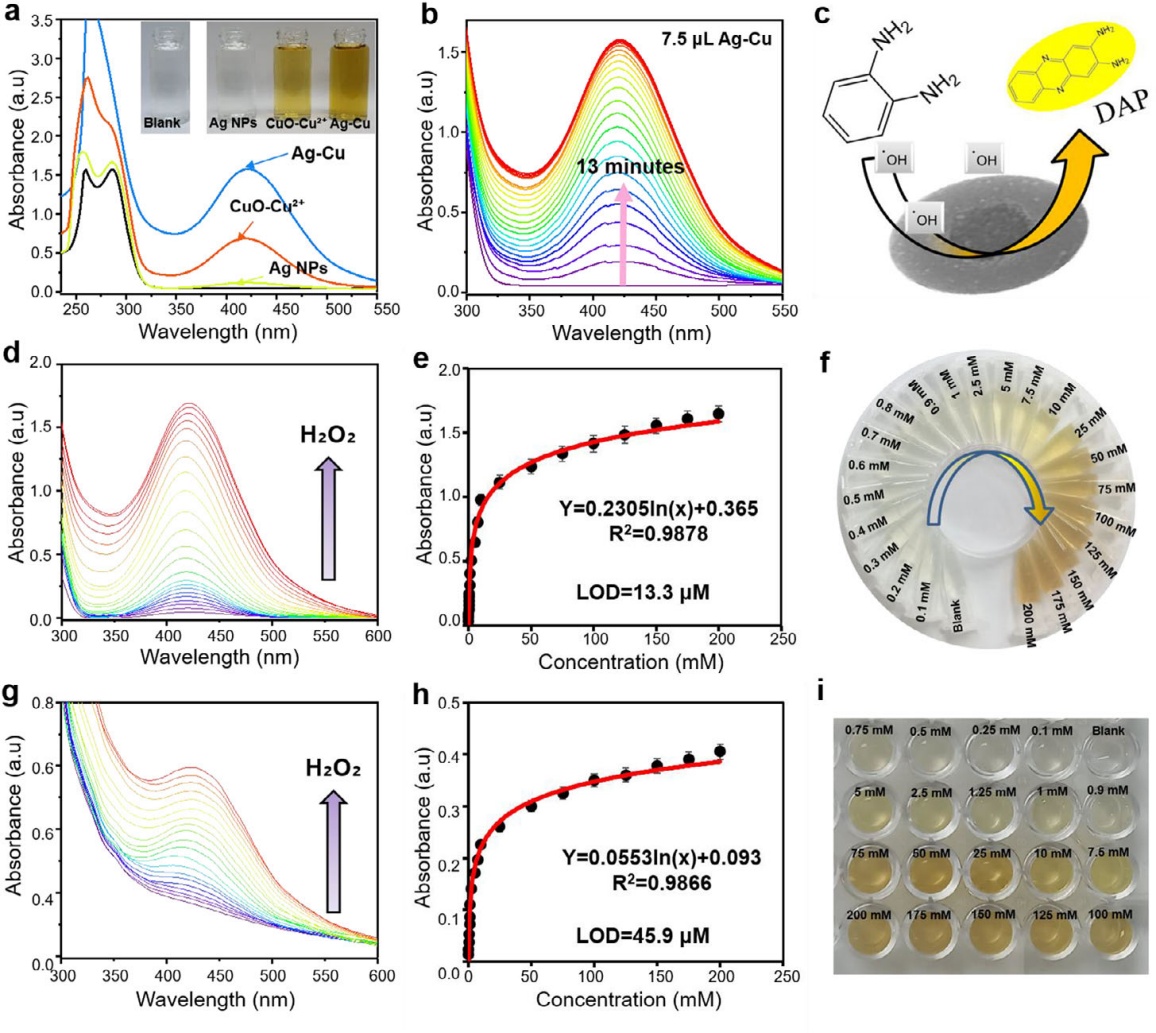
a) Ultraviolet—visible (UV–Vis) absorption spectra of o‐phenylene diamine (OPD)/H_2_O_2_ mixtures without and with different catalysts: Ag nanoparticles (NPs), Ag–Cu, and CuO–Cu^2^⁺. Inset photos show the corresponding color response. b) UV–Vis/time regarding the OPD conversion to DAP via 7.5‐µL Ag–Cu bimetallic nanoenzyme. c) Mechanism of OPD peroxidation in 2,3‐diaminophenazine (DAP) using the synthesized Ag–Cu bimetallic. d) UV–Vis response of OPD and Ag–Cu nanoenzyme mixture to H_2_O_2_ assay. e) Corresponding nonlinear response to H_2_O_2_ concentrations up to 200 × 10^−3^ m at *λ* = 417 nm (mean ± SD, *n* = 3). f) Colorimetric response of OPD and Ag–Cu nanoenzyme mixture to different H_2_O_2_ concentrations. g) UV–Vis response of Ag–Cu nanoenzyme‐based solid kit to various H_2_O_2_ concentrations up to 200 × 10^−3^ m. h) Corresponding nonlinear response of solid kit to H_2_O_2_ concentrations up to 200 × 10^−3^ m (mean ± SD, *n* = 3). i) Corresponding colorimetric response of Ag–Cu nanoenzyme‐based solid kit to various H_2_O_2_ concentrations up to 200 × 10^−3^ m.

Catalytic materials with enzyme‐like activity have been developed to overcome the limitations of natural enzymes, such as poor stability and high cost.^[^ ^84^ ^]^ Ag–Cu BMNPs not only exhibit catalytic reduction activity but also possess intrinsic peroxidase‐mimic activity, enabling the oxidation of OPD into DAP in the presence of H₂O₂, producing an orange‐yellow color (**Figure** **5**a). Without a catalyst, no peak is observed at *λ* = 415 nm (Figure 5a: black curve), indicating that peroxidation of OPD does not occur due to the high kinetic barrier.^[^ ^36^ ^]^ Figure 5a shows the superior peroxidase activity of Ag–Cu BMNPs compared with Ag NPs and CuO–Cu^2^⁺. Ag NPs displayed minimal peroxidase activity (yellow curve), whereas CuO–Cu^2^⁺ exhibited weak activity, requiring extended time to oxidize OPD into DAP (orange curve). Meanwhile, Ag–Cu BMNPs exhibited higher peroxidase‐mimic activity, underscoring the enhanced catalytic performance of the bimetallic structure over monometallic counterparts (blue curve), as further demonstrated in Videos S4 and S5 (Supporting Information).^[^ ^85^ ^]^ The peroxidase activity of Ag–Cu BMNPs was evaluated using various doses (5–7.5 µL). Experimental results (Figure 5b and Figure S15, Supporting Information) indicated that increasing the NP dose enhanced the peroxidase‐mimic activity and reduced the time required for OPD conversion to DAP from 60 to 13 min. The maximum activity was observed with 7.5‐µL Ag–Cu, achieving a peak intensity of 1.69 at *λ* = 415 nm in 13 min (Figure 5b), compared with 1.1 a.u. at 60 min for 5‐µL Ag–Cu (Figure S15, Supporting Information). This rapid oxidation is attributed to increased active sites on the catalyst, enhancing the OH^•^ production.^[^ ^36^ ^]^ In comparison, single‐component Ag NPs and CuO–Cu^2^⁺ exhibited markedly lower catalytic activity, even at high doses. For instance, 20‐µL Ag NPs required 60 min to achieve a peak intensity of 0.36 a.u. (Figure S16, Supporting Information) whereas 20‐µL CuO–Cu^2^⁺ took 30 min to reach 1.1 a.u. (Figure S17, Supporting Information), highlighting the superior peroxidase‐mimic activity of Ag–Cu BMNPs.

The enzymatic activity of Ag–Cu BMNPs was demonstrated through H_2_O_2_ detection using both colorimetric (Figure 5) and electrochemical methods (Figure S18, Supporting Information). Ag–Cu NPs, in combination with OPD, served as a solution/solid‐based colorimetric probe for H_2_O_2_ detection (Figure 5d,g). Optimal conditions, including NP dose, OPD concentration, and incubation time, were determined based on previous results (Figure 5a and Figure S14, Supporting Information). UV–Vis analysis showed a gradual increase in peak intensity at *λ* = 415 nm as H_2_O_2_ concentration increased to 200 × 10^−3^ m (Figure 5d), with a corresponding color change from colorless to orange‐yellow, indicating DAP formation (Figure 5f). Figure 5e shows a nonlinear response from 0 to 200 × 10^−3^ m, following the equation *Y* = 0.2305ln(x) + 0.365, with a high correlation coefficient (*R* ^2^ = 0.987). The LOD was calculated as 13.3 × 10^−6^ m based on the signal‐to‐noise ratio^[^ ^86^ ^]^:

(2) $$\frac{\mathrm{Signal}\;\mathrm{intensity}\;A}{\mathrm{Concentration}\;A}=3.3\delta /\;\mathrm{LOD}$$

where δ denotes the blank standard deviation, and *A* denotes the lowest measured concentration.

To enhance practical applicability, both OPD and Ag–Cu BMNPs were embedded in agarose hydrogel, creating a portable solid kit for H_2_O_2_ detection. This solid sensor demonstrated a colorimetric response, with an increased peak intensity at *λ* = 415 nm as H_2_O_2_ concentration increased to 200 × 10^−3^ m (Figure 5g), mirroring the response seen in the solution‐based sensor (Figure 5i). The response plot (Figure 5h) followed a nonlinear dynamic from 0 to 200 × 10^−3^ m, modeled by *Y* = 0.0553ln(x) + 0.093, with an LOD of 45.9 × 10^−6^ m. These findings confirm the exceptional peroxidase‐mimic activity of Ag–Cu BMNPs, making them effective for both solution and solid‐state H_2_O_2_ sensing. This approach could be extended to detect other biomolecules, such as glucose, lactate, uric acid (UA), and cholesterol, using an enzymatic strategy.

#### 2.4.3 Ag–Cu NPs as Electrocatalytic Glucose Sensor

**Figure 6:**
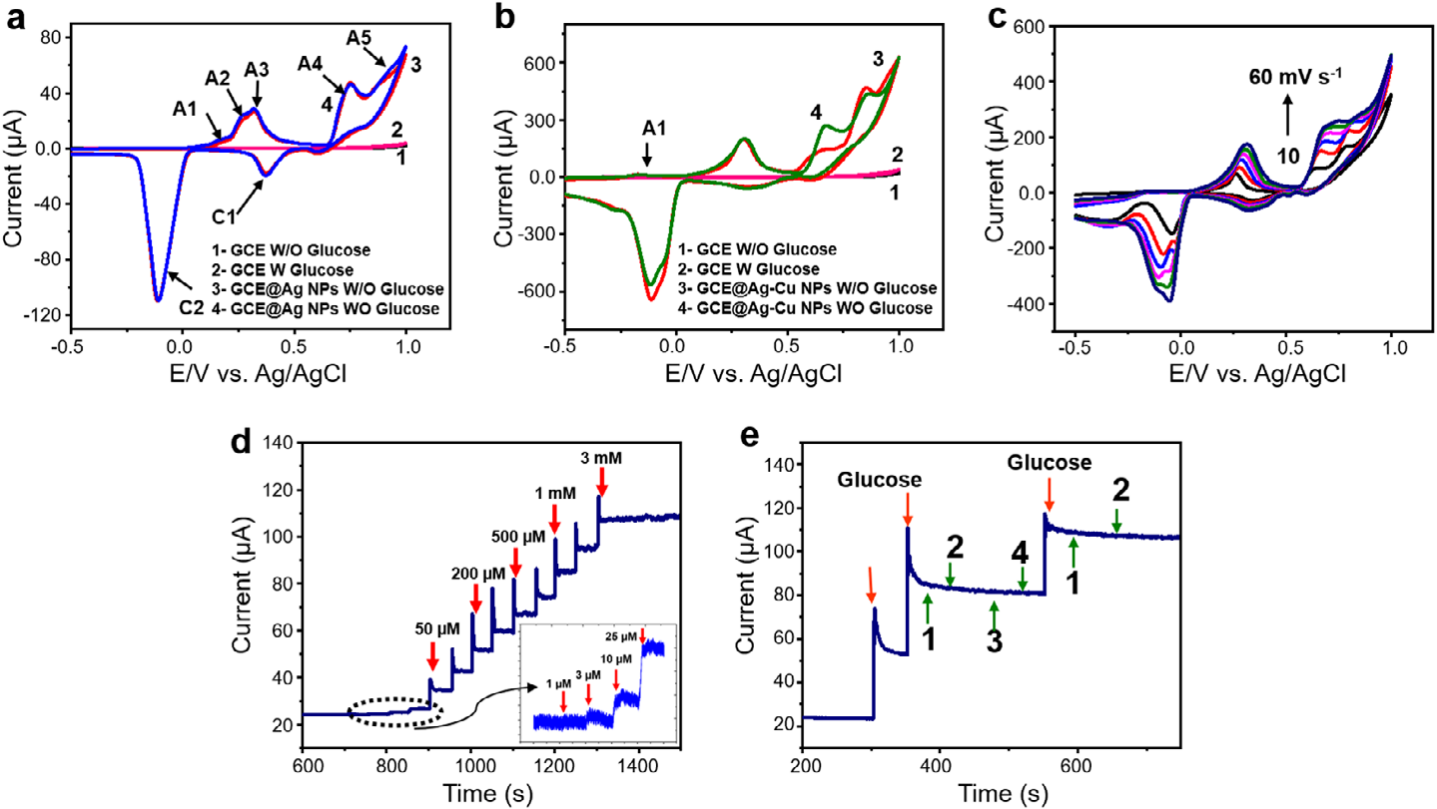
Electrochemical responses of various electrodes toward glucose oxidation in 0.1 m NaOH. a) Cyclic voltammograms (CVs) for bare glassy carbon electrode (GCE) (Curves 1 and 2) and GCE@Ag (Curve 3 and 4). b) CVs for GCE@Ag–Cu in the absence and presence of 1 × 10^−3^ m glucose at a scan rate of 50 mVs^−1^. c) CVs of GCE@Ag–Cu for 1 × 10^−3^ m glucose at different scan rates (10–60 mVs^−1^). d) amperometric response of GCE@Ag‐Cu for various glucose concentrations (1 × 10^−6^ m to 3 × 10^−3^ m) in 0.1‐m NaOH, e) selective determination of glucose for GCE@Ag–Cu in the presence of interferences, such as (1) uric acid (UA), (2) dopamine hydrochloride (DA), (3) ascorbic acid (AA), and (4) urea.

The electrocatalytic activity of a glassy carbon electrode (GCE) modified with Ag–Cu BMNPs was investigated for the nonenzymatic oxidation of glucose. The modification methods of the GCE are detailed in Supporting Information (Chapter S9, Supporting Information). **Figure** **6**a shows the cyclic voltammograms (CVs) for GCE@Ag in 0.1‐m NaOH, recorded before and after 1 × 10^−3^ m glucose addition. Curves 1 and 2 represent the responses of bare GCE before and after glucose addition at a scan rate of 50 mVs^−1^, demonstrating no catalytic activity toward glucose oxidation. Curve 3 in Figure 6a displays the electrochemical behavior of GCE@Ag in NaOH, indicating five anodic peaks (A1–A5) corresponding to the electrochemical reactions of Ag. A1 is related to the electrodissolution of Ag as [Ag(OH)₂]⁻, whereas A2 and A3 represent the formation of monolayer and multilayer Ag₂O. A4 indicates the oxidation of Ag and Ag₂O to AgO, and A5 corresponds to the formation of Ag₂O₃.^[^ ^87^, ^88^, ^89^ ^]^ The addition of glucose did not alter the electrochemical response of GCE@Ag (Figure 6a: Curve 4), indicating that Ag NPs are inactive for nonenzymatic glucose oxidation. To assess the catalytic activity of Ag–Cu BMNPs, similar experiments were conducted with an Ag–Cu BMNP‐based electrode (GCE@Ag–Cu; Figure 6b). Curves 1 and 2 show the electrode response in the absence and presence of glucose, respectively, confirming no activity from bare GCE. Curve 3 shows the response of GCE@Ag–Cu in NaOH, where peak A1 corresponds to the dissolution of Cu as Cu(OH)₂^[^ ^90^ ^]^ and the remaining peaks reflect Ag oxide formation. Notably, GCE@Ag–Cu displayed electrocatalytic activity toward glucose oxidation at +0.65 V (Figure 6b: Curve 4), attributable to Cu, known for facilitating glucose electro‐oxidation.^[^ ^91^, ^92^ ^]^

Figure 6c shows the CVs of GCE@Ag‐Cu in 100 × 10^−3^ m NaOH containing 1 × 10^−3^ m glucose at varying scan rates (10–60 mV s⁻¹). The oxidation peak current of glucose and the anodic and cathodic peak currents increased linearly with the scan rate, indicating that glucose oxidation on Ag–Cu follows a diffusion‐controlled mechanism (*R* ^2^ = 0.991). The Cu NPs play a crucial role in the glucose oxidation pathway, where Cu(II) is converted to Cu(III), which then catalytically converts glucose to gluconic acid and H₂O₂.^[^ ^93^ ^]^ The addition of Ag NPs enhances the catalytic activity of the Cu‐based system, as previously reported. Figure 6d shows the amperometric current–time (*I–t*) curve for glucose oxidation at GCE@Ag–Cu in a stirred 0.1‐mNaOH solution at +0.7 V. The sensor demonstrated a dynamic detection range of 1 × 10^−6^ m to 3 × 10^−3^ m, with each glucose addition resulting in an immediate current increase. A linear relationship was observed between glucose concentration and current (*R* ^2^ = 0.996), with an LOD of 0.1 × 10^−6^ m (S/N = 3) and a sensitivity of 5221 µA × 10^−6^ m⁻¹ cm⁻^2^. Performance comparisons with previously reported sensors are provided in Table S4 (Supporting Information). Selectivity was tested against potential interfering biomolecules, such as UA, l‐ascorbic acid (l‐AA), dopamine hydrochloride (DA), and urea. As shown in Figure 6e, GCE@Ag‐Cu maintained a stable current response to 500 × 10^−6^ m glucose, even after the addition of 10 × 10^−3^ m interferents, demonstrating high specificity and anti‐interference capability, thereby confirming GCE@Ag‐Cu's practical applicability for glucose detection.

## 3 Conclusion

In summary, this work presents a novel, sunlight‐mediated synthesis method for Ag–Cu BMNPs that is ecofriendly, has low energy input, and occurs rapidly at room temperature. Using sunlight as the reducing agent and a CTAB/GPEOL surfactant mixture as both a coreducing and capping agent, Ag⁺ was reduced to Ag, which then mediated the reduction of Cu^2^⁺ to Cu via activated species from water radiolysis. The novel method facilitated the creation of two types of reusable catalytic membrane reactors, PTFE@Ag–Cu and ACF@Ag–Cu, which demonstrated high efficiency in pollutant treatment and catalyst recollection, considerably lowering treatment costs and toxicity. Excellent reusability is showcased as PTFE@Ag–Cu converts 2100 mL of 0.15 × 10^−3^ m *p*‐NP to *p*‐AP over 105 cycles while ACF@Ag–Cu achieves a similar conversion for 1500 mL over 75 cycles. In addition, the Ag–Cu BMNPs exhibit enzymatic activity, allowing for effective colorimetric H₂O₂ detection and electrochemical glucose detection. Such a sustainable synthesis method presents a versatile platform for BMNP preparation, with promising applications in pollutant treatment and biosensing, offering a cost‐effective alternative to conventional methods.

## 4 Experimental Section

### Chemical Preparations

All materials involved in this study were used directly as purchased without further purification. 1‐bromododecane (97%), agarose, CTAB (98%), sodium borohydride (NaBH_4_, 98%), potassium chloride (NaCl, ACS 99%), OPD (99.5%), hydrogen peroxide solution (H_2_O_2_, 30%), agarose (Type I, low EEO), UA (99%), l‐AA (99%), DA, urea (ACS, 99%), sodium chloride (NaCl, ACS 99%), phosphate buffer solution, and glucose (99.5%) were purchased from Sigma–Aldrich Chemicals Co., Inc. (South Korea).

### Synthesis of Gemini Nonionic Surfactant

GPEOL was prepared using a method described in our previous report.^[^ ^94^ ^]^ The detailed synthetic procedure is shown in Figure S19 (Supporting Information).

### Characterizations of Synthesized Nanocomposites

The synthesis of Ag‐Cu BMNPs was achieved using a modified coreduction approach (Supporting Information). The chemical structure of GPEOL was confirmed using both FTIR and ^1^H NMR spectra (Figures S20–S22, Supporting Information).

### Instruments

The FTIR and ^1^HNMR spectra were used to assess the predicted chemical structure of the prepared hydroxyphenyl aminopropyl cationic surfactant. The FTIR spectroscopy was performed using Bench top 961 ATI Mattsonm integrated with Win First V2.01 Software. The ^1^HNMR spectra were assigned using a Bruker FT‐NMR spectrometer (Burker, Avance III 400 MHz). The morphologies of the prepared nanocomposites were scanned via TEM (Joel JeM–2100) and SEM (model JSM7500F) at an accelerating voltage of 15 kV. The UV–Vis spectroscopy was conducted using a Shimadzu UV‐2550 device to investigate the surface plasmon resonance of the synthesized nanocomposites and monitor their catalytic activity.

### Catalytic Activity

The catalytic performances of the synthesized Ag‐Cu BMNPs, Ag NPs, and CuO–Cu^2^⁺ were investigated for *p*‐NP transformation using NaBH_4_ as a reducing agent.^[^ ^74^ ^]^ For this purpose, different concentrations (1–6 µL) of each synthesized catalyst were individually injected into a 50‐mL solution containing different concentrations of *p*‐NP and freshly prepared NaBH_4_. Two different concentrations of *p*‐NP were examined, with 1 and 1.5 of *p*‐NP (5 × 10^−3^ m) mixed with 49 m and 48.5 mL of NaBH_4_ (20 × 10^−3^ m), respectively. Subsequently, the different doses (1–6 µL) of the catalysts were separately added to the p‐NP/NaBH_4_ mixtures. After a specific time, the remaining *p*‐NP concentration was determined by measuring the UV–Vis absorbance intensity using a UV–Vis spectrophotometer.

### Peroxidase Nanoenzyme Activity

The peroxidase‐mimic activities of the Ag–Cu BMNPs, Ag NPs, and CuO–Cu^2^⁺ were investigated by studying their ability to catalyze the conversion of OPD to DAP in the presence of H_2_O_2_.^[^ ^36^ ^]^ Various concentrations were examined, with different doses of the synthesized catalysts being added separately into a 50‐mL aqueous solution of H_2_O_2_ and OPD with final concentrations of 100 and 0.5 × 10^−3^ m, respectively. The DAP concentration was measured by recording the UV–Vis absorbance intensity at *λ* = 417 nm at specific time intervals.

### Colorimetric Solution/Solid H_2_O_2_ Detection

The Ag–Cu bimetallic catalyst was used as an artificial peroxidase enzyme for colorimetric H_2_O_2_ assay in both solution and solid sensing platforms. OPD was used as a peroxidase substrate that produces DAP with an orange‐yellow color in the presence of H_2_O_2_ and peroxidase enzyme. For the colorimetric H_2_O_2_ detection in solution, a stock solution comprising 40‐µL Ag–Cu enzyme with 50‐mL OPD (0.5 × 10^−3^ m) was prepared as a sensor probe. Different H_2_O_2_ concentrations (100 µL) were added to the sensor probe solution (900 µL), and the UV–Vis response was recorded at *λ* = 417 nm after a 20‐min incubation period.

To broaden the sensor applicability, a portable solid colorimetric H_2_O_2_ sensor was constructed by integrating OPD and Ag–Cu enzymes into an agarose hydrogel structure. First, agarose hydrogel solution (8 mg mL^−1^) was prepared by dissolving a specified quantity of agarose in boiled water, and the temperature was readjusted to 55 °C. Then, 1 mL OPD (1 × 10^−3^ m) and 1 mL agarose hydrogel were mixed at 55 °C. Afterward, 10 µL Ag–Cu was added to the mixture. Subsequently, 70 µL of the previous sensor probe solution was transferred to each well of a 96‐microplate and allowed to solidify at 25 °C before storing at −4 °C for further sensing application. Finally, 70 µL of different H_2_O_2_ concentrations was injected into each well, and the UV–Vis response was recorded at *λ* = 417 nm after a 20‐min incubation period using a microplate reader.

### Toxicity Test

The toxicity of the samples was determined using the standard MTT protocol according to ISO 10993–5:2009 on L‐929 fibroblast cells (details are provided in Supporting Information). The Ag, CuO–Cu^2^⁺, and Ag–Cu catalysts were tested at concentrations of 0.1 and 0.2 µL mL^−1^, which are higher than those used for catalytic activity (6 µL/50 mL dye). In addition, the MTT assay was used to check whether the Ag–Cu bimetallic structure could be detached from the synthesized catalytic membranes during the recycling test. Distilled water was injected through both synthesized membranes individually, and then the outlet water toxicity was examined via MTT assay.

### Statistical Analysis

In this current study, data are presented as mean ± SD.

## Conflict of Interest

The authors declare no conflict of interest.

## Supporting information



Supporting Information

Supplemental Video 1

Supplemental Video 2

Supplemental Video 3

Supplemental Video 4

Supplemental Video 5

## Data Availability

The data that support the findings of this study are available from the corresponding author upon reasonable request.
