# Supplementary material for: Ecofriendly Sunlight‐Mediated Nontoxic Bimetallic Nanoparticles: Synthesis, Reusable Catalytic Membrane, and Biosensor Applications
Source: Adv Sci (Weinh). 2025 Apr 30;12(27):2503120. doi: 10.1002/advs.202503120 (PMC12279182; doi:10.1002/advs.202503120)
Supplement: Supplementary file 1 — Supporting Information [file ADVS-12-2503120-s002.docx]

**Supporting Information**

**Ecofriendly Sunlight-Mediated Nontoxic Bimetallic Nanoparticles: Synthesis, Reusable Catalytic Membrane, and Biosensor Applications**

Samy M. Shaban^1,2,3^, Sihyeok Kim^4^, N. M. El Basiony^1^, Jincymol Kappen^1^, Mohamed H. Mostafa^1^, Ahmed Y. Elbalaawy^1^, Mohamed R. Elmasry^1^, Jihoon Shin^1,2^, Il Jeon^4,5,6^*, Dong-Hwan Kim^1,2^*

^1^School of Chemical Engineering, Sungkyunkwan University (SKKU), Suwon 16419, Republic of Korea.

^2^Biomedical Institute for Convergence at SKKU (BICS), Sungkyunkwan University (SKKU), Suwon 16419, Republic of Korea

^3^Egyptian Petroleum Research Institute, Nasr City 11727, Cairo, Egypt.

^4^Department of Nano Engineering, Department of Nano Science and Technology, SKKU Advanced Institute of Nanotechnology (SAINT), Sungkyunkwan University (SKKU), Suwon 16419, Republic of Korea

^5^SKKU Global Research Center (SGRC), Sungkyunkwan University (SKKU), Suwon 16419, Republic of Korea

^6^New Industry Creation Hatchery Center(NICHe), Tohoku University Sendai, 980-8576 Japan

*Email: il.jeon@spc.oxon.org, dhkim1@skku.edu

# Contents

1. Ag–Cu BMNPs, Ag NPs, and CuO–Cu²⁺ Syntheses
2. *p*-NP conversion Comparison Among Ag–Cu BMNPs, Ag NPs, and CuO–Cu²⁺
3. More Detailed Investigation on Catalytic Reaction Kinetics
4. Reusability Test for PETE@Ag–Cu and ACF@Ag–Cu Catalytic Membranes
5. Toxicity Test for Ag NPs, CuO–Cu²⁺, Ag–Cu BMNPs
6. Comparison of UV-vis/Time Data for OPD Conversion to DAP Using Ag–Cu BMNPs, Ag NPs, and CuO–Cu²⁺
7. Detailed Mechanism for Peroxidase Activity
8. Preparation Method of Modified GCE using Ag–Cu BMNPs
9. H_2_O_2_ and Glucose Detection Using GCE@Ag–Cu
10. Synthesis of Gemini Nonionic Amphiphiles
11. Fabrication of Ag-Cu bimetallic using individual and mixed surfactant of CTAB and GPEOL (Video S1).
12. Performance of PTFE@Ag–Cu Catalytic Membrane in Converting p-NP to p-AP (Video S2)
13. Performance of ACF@Ag–Cu Catalytic Membrane in Converting p-NP to p-AP (Video S3)
14. Peroxidase-Mimic Activity of Ag–Cu BMNPs (5 μL; Video S4)
15. Peroxidase-Mimic Activity of Ag–Cu BMNPs (7.5 μL; Video S5)
16. **Ag–Cu BMNPs, Ag NPs, and CuO–Cu²⁺ Syntheses**

Ag–Cu BMNPs were synthesized via a modified coreduction method using sunlight as a green and sustainable source of reducing agent. First, 25-mL CTAB aqueous solution (2 mM) and 25-mL GPEOL aqueous solution (500 μM) were mixed well under stirring for 5 min. To this solution, 50-mL AgNO_3_ aqueous solution (50 mM) was added, followed by the addition of 50-mL Cu(NO_3_)_2_ aqueous solution (200 mM) under stirring for another 5 min. The resulting solution was exposed to sunlight, and the solution color changed from aqua blue to brownish green after 10 min of exposure (**Figure 1a**). The detailed synthesis mechanism of Ag-Cu BMNPs is shown in figure S1a. For comparison, Ag NPs were prepared following the same procedure but without the addition of the Cu precursor, resulting in a brownish-red solution, indicating the formation of Ag NPs. In addition, a control experiment was repeated without the Ag precursor; however, no change in the solution color was observed before and after exposure to sunlight for several weeks, indicating the difficulty in synthesizing Cu NPs (**Figure S2**). As seen from SEM (**Figure S3a**) and TEM (**Figure S3c**), Ag NPs with a spherical structure were successfully prepared. The d-spacing of crystal planes of Ag NPs were calculated from the inset TEM images (**Figure S3c**) as 0.23 and 0.19 nm, corresponding to the [111] and [200] planes, respectively. The TEM image (**Figure S3d**) reflects the presence of CuO NPs, with a d-spacing value of 0.24 nm.


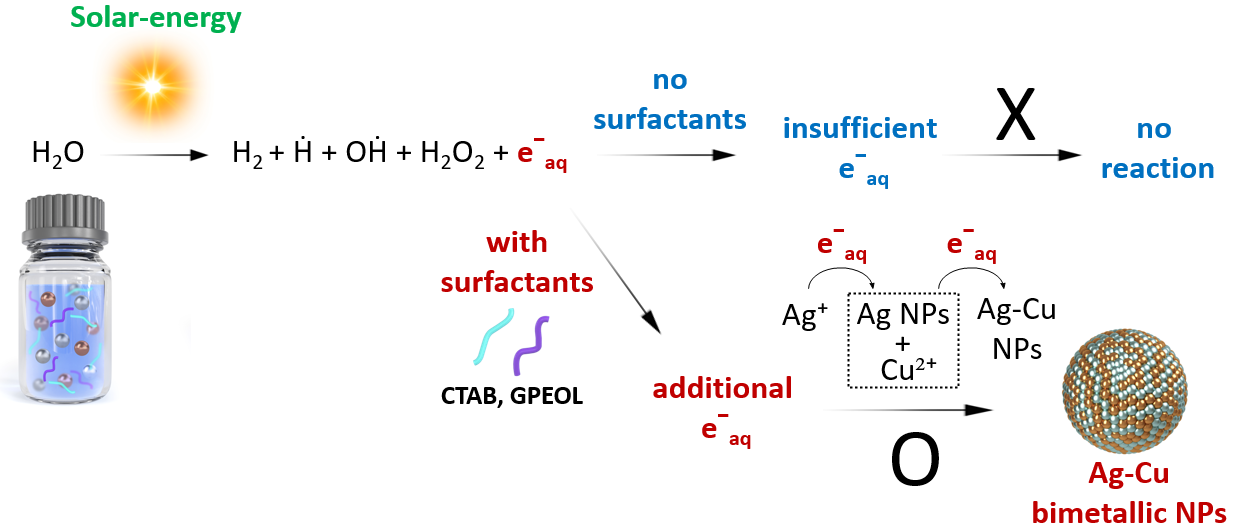


**Figure S1.** Detailed synthesis mechanism of Ag-Cu BMNPs and the role of CTAB and GPEOL surfactants.


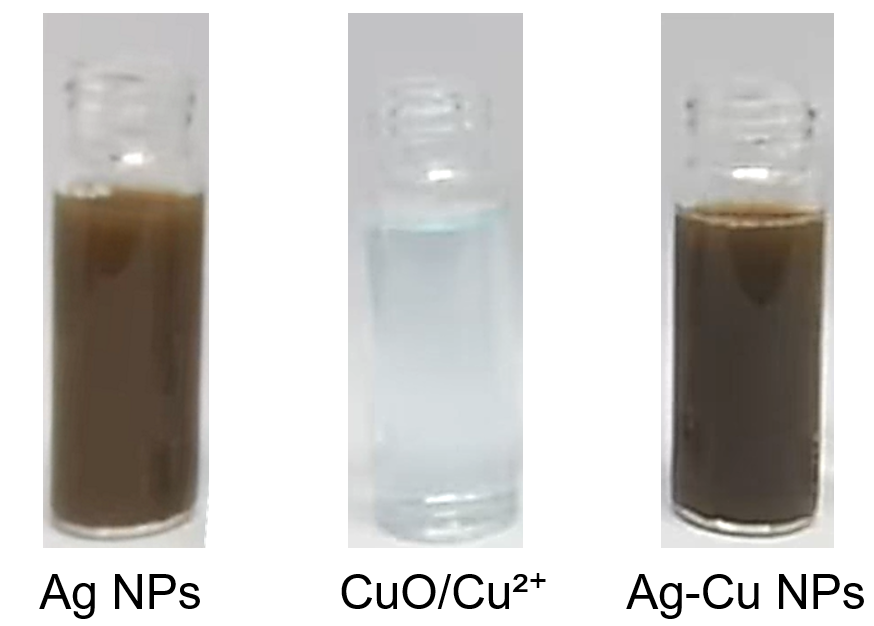


**Figure S2.** Image of Ag NPs, CuO–Cu²⁺, and Ag–Cu BMNPs-dissolved solutions


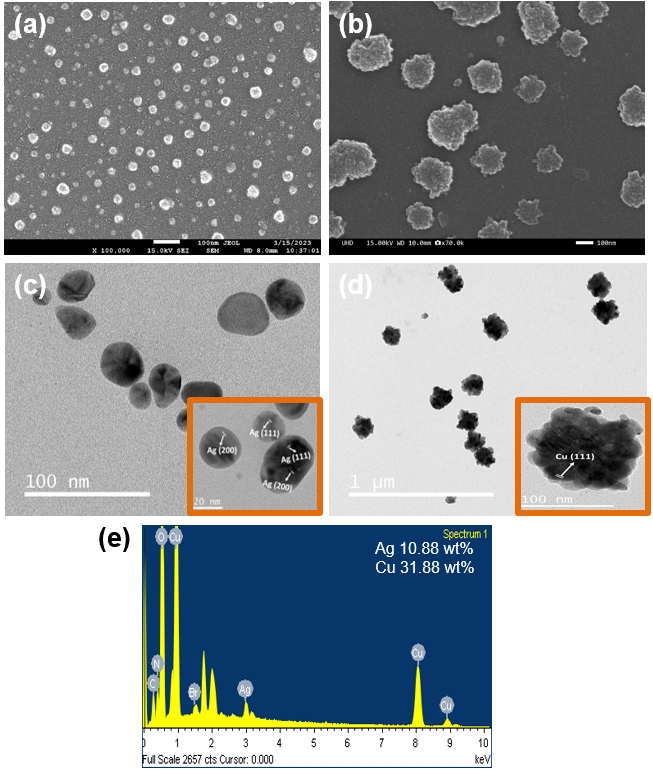


**Figure S3.** SEM images of (a) Ag NPs and (b) CuO–Cu²⁺; TEM images of (c) Ag NPs and (d) CuO–Cu²⁺; (e) chemical composition of Ag–Cu BMNPs confirmed by EDS.

Figure S4: SEM of the micro-sized domain of synthesized Ag-Cu with different magnification power shown individual nanoparticles.


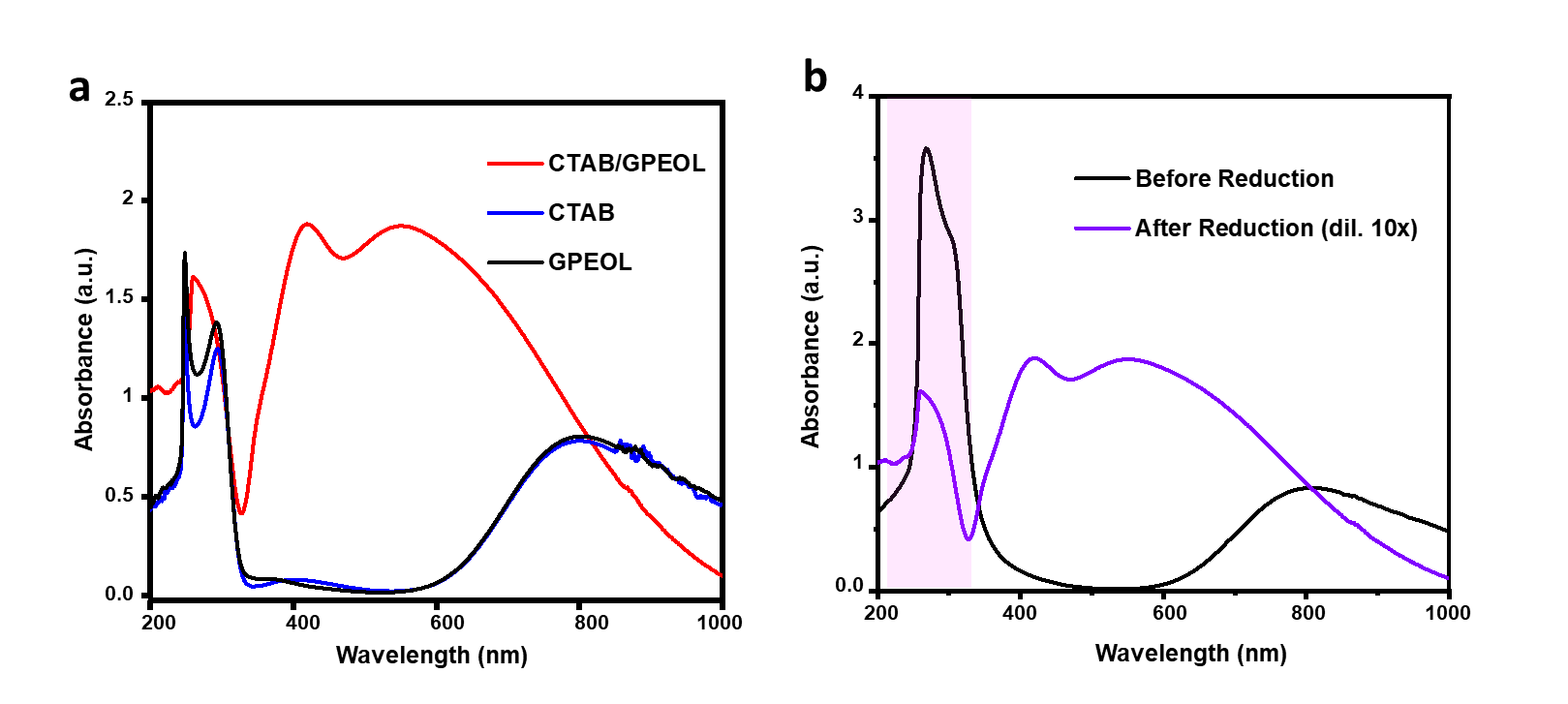


**Figure S5**: (a) UV-Vis spectra of Ag-Cu fabrication using a binary surfactant mixture (CTAB and GPEOL) compared to individual surfactants (CTAB or GPEOL). (b) UV-Vis spectra showing the variation in the absorbance peak of the binary surfactant mixture at 250 nm before and after the reduction process under sunlight.

1. ***p*-NP Conversion Comparison Among Ag–Cu BMNPs, Ag NPs, and CuO–Cu²⁺**


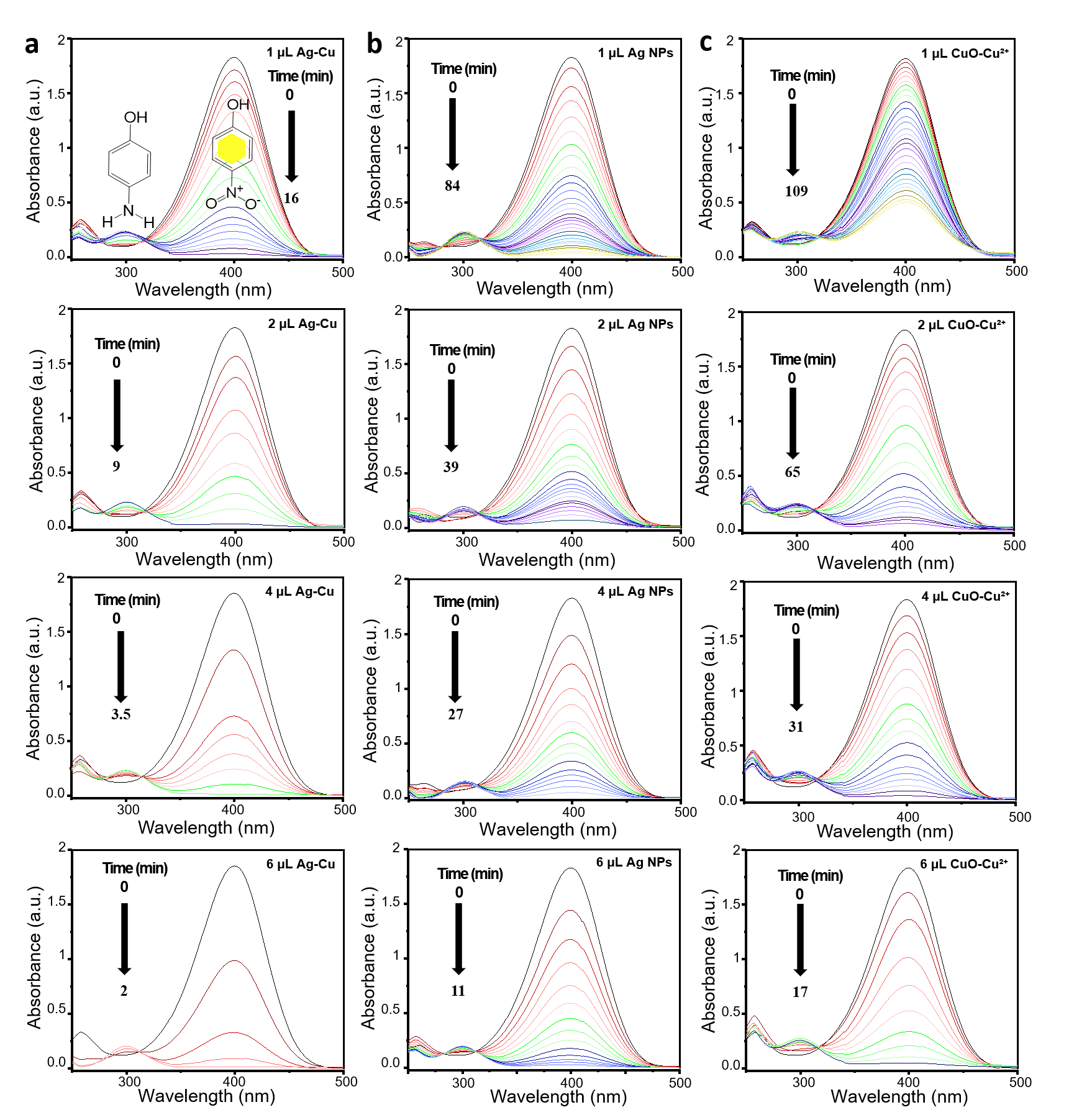


**Figure S6.** UV-vis spectra of the reduction of 0.1-mM *p-*NP to *p****-***AP using various doses of the synthesized catalysts: (a) Ag–Cu BMNPs, (b) Ag NPs, and (c) CuO–Cu²⁺.


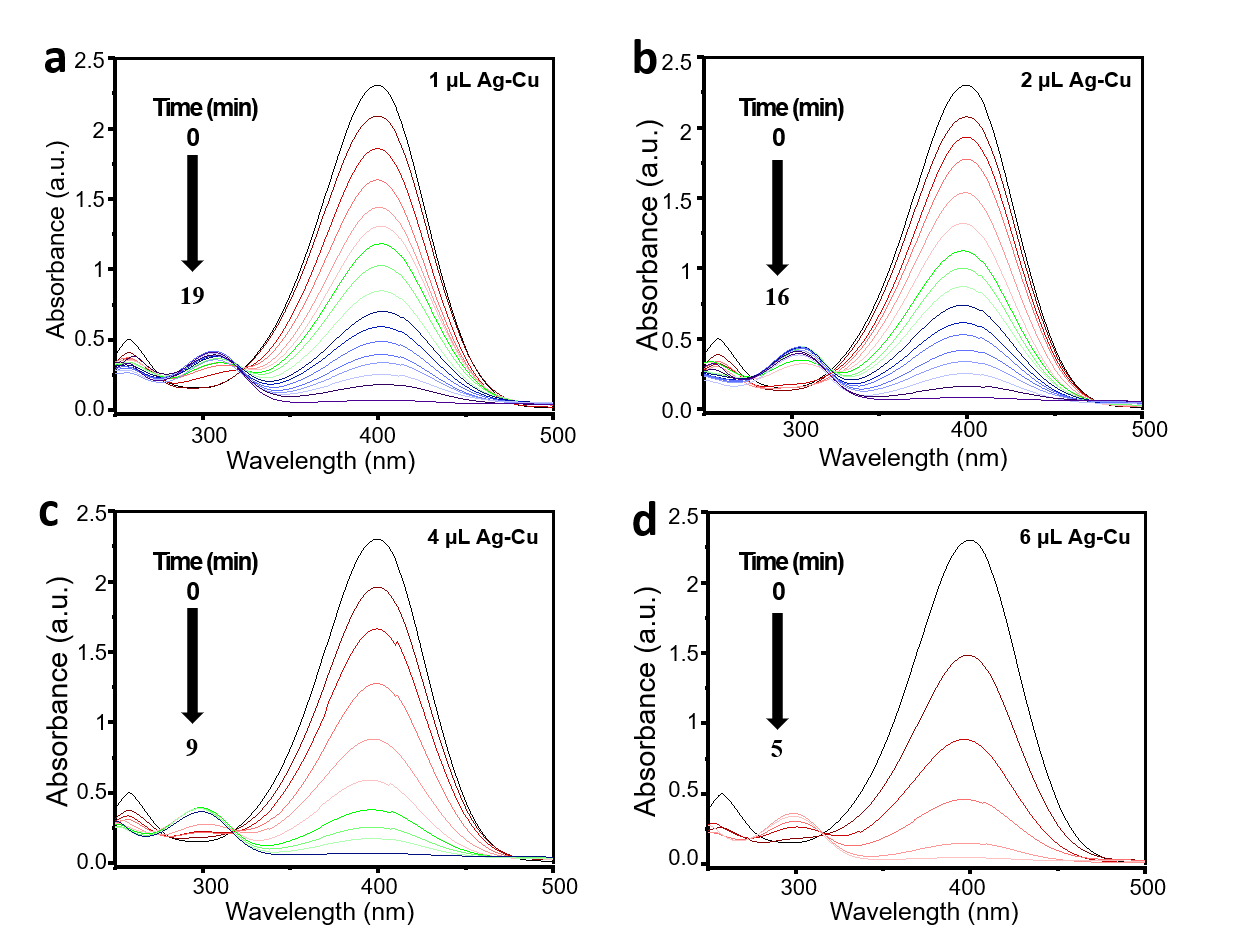


**Figure S7.** UV-vis absorption spectra showing the catalytic reduction of 0.15-mM *p*-NP to *p*-AP using different doses of Ag–Cu BMNPs.


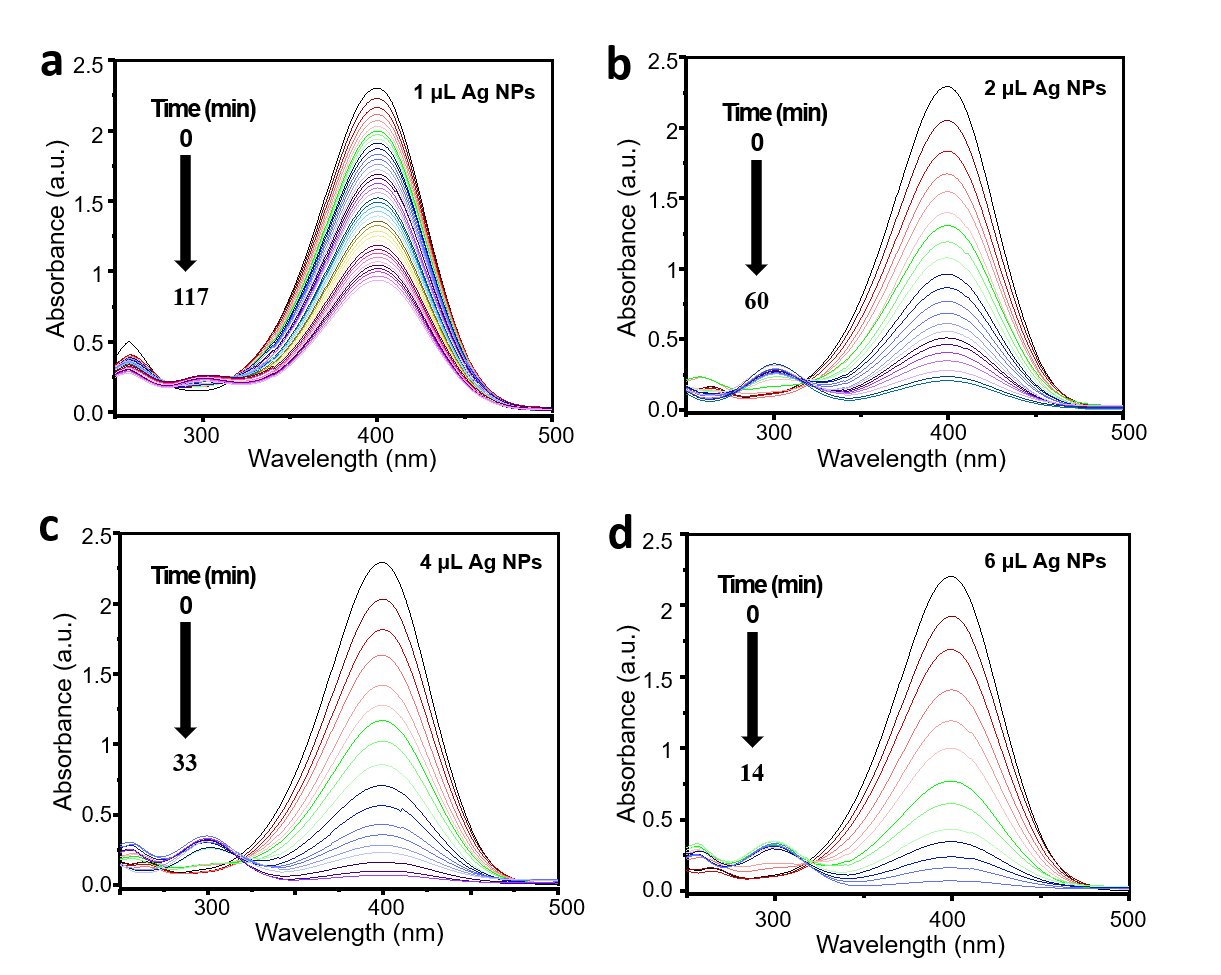


**Figure** **S8.** UV-vis absorption spectra showing the catalytic reduction of 0.15-mM *p*-NP to *p*-AP using different doses of Ag NPs.


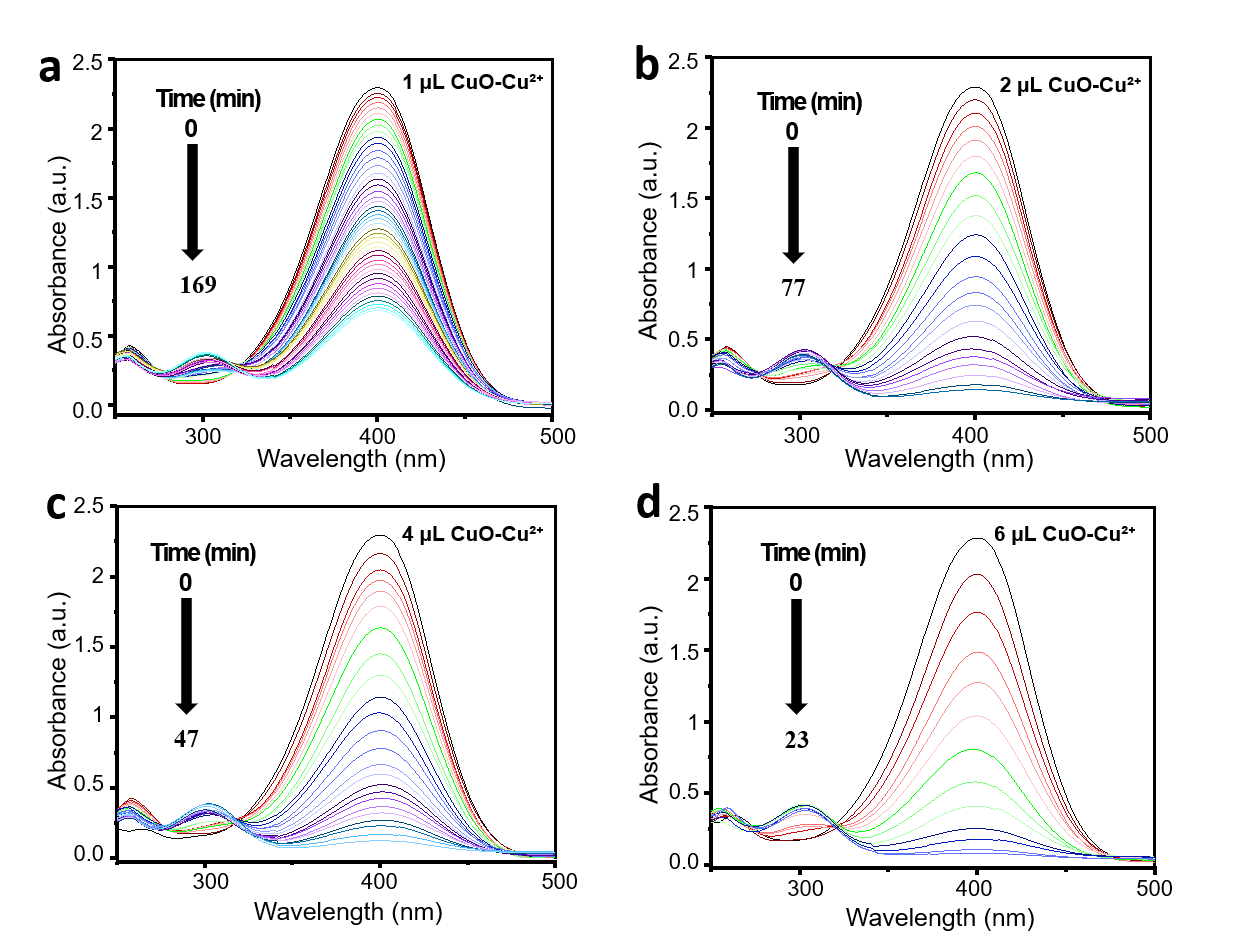


**Figure S9.** UV-vis absorption spectra showing the catalytic reduction of 0.15-mM *p*-NP to *p*-AP using different doses of CuO–Cu²⁺.


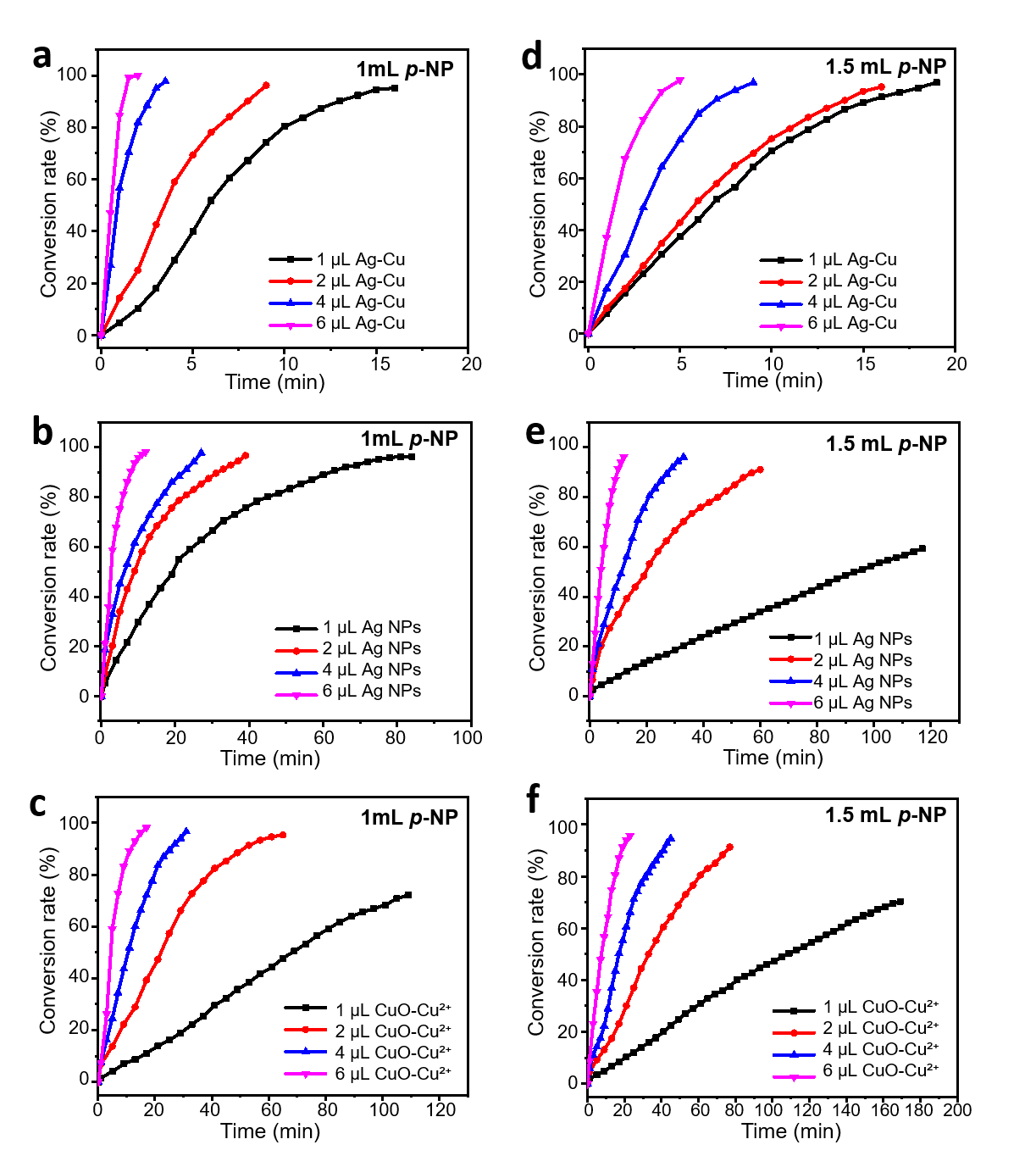


**Figure S10.** (a–c) Conversion rate of the reduction of 0.1-mM *p-*NP to *p-*AP in the presence of NaBH_4_ as a reducing agent using different doses of Ag–Cu BMNPs, Ag NPs, and CuO–Cu²⁺; (d–f) conversion rate of the reduction of 0.15-mM *p-*NP to *p-*AP in the presence of NaBH_4_ as reducing agent using different doses of Ag–Cu BMNPs, Ag NPs, and CuO–Cu²⁺.

1. **More Detailed Investigation on Catalytic Reaction Kinetics**

The kinetics rate constant ($k_{\mathrm{app}}$) was assessed using Equation S1, following the pseudo-first-order kinetic model, as the NaBH_4_ concentration was high to ensure that it remains fixed during *p*-NP reduction [1]:

$\ln\left( \frac{C_{t}}{C_{o}} \right)=-k_{\mathrm{app}}t$, **(S1)**

where C_t_/C_o_ (*p*-NP concentration at time t/concentration at zero time) was calculated based on the UV-vis data that equated to the A_t_/A_o_ ratio according to the Beer–Lambert law. Thus, Equation S1 can be rewritten as follows:

$\ln\left( \frac{C_{t}}{C_{o}} \right)=ln\left( \frac{A_{t}}{A_{o}} \right)=-k_{\mathrm{app}}t$. **(S2)**

Kinetically, the reduction of *p*-NP using Ag–Cu BMNPs, Ag NPs, and CuO–Cu²⁺ followed a pseudo-first-order model (**Figure S9**). The data clearly indicate a dose-dependent catalytic activity. The extracted $k_{\mathrm{app}}$ values are listed in **Table S1**, demonstrating catalytic activity-dose dependency and the considerable impact of Ag and Cu doping over monometallic NPs. For Ag–Cu concentrations of 1, 2, 4, and 6 μL, the $k_{\mathrm{app}}$ values were 0.2071, 0.3225, 1.065, and 2.28 min^−1^, respectively, for 0.1-mM *p*-NP conversion to *p*-AP (**Table S1**), attributable to an increasing number of active sites of the specified catalyst, thereby maximizing the total surface area.

Under the same concentration and operating conditions, Ag–Cu BMNPs exhibited enhanced catalytic activity over the monometallic nanostructures, namely, Ag NPs and CuO–Cu²⁺, as indicated by the extracted $k_{\mathrm{app}}$ values (**Table S1**). The k_app_ values regarding 0.1-mM *p*-NP conversion to *p*-AP at a fixed dose of 6 μL were 2.28, 0.3045, and 0.1902 min^−1^ for Ag–Cu BMNPs, Ag NPs, and CuO–Cu²⁺ respectively. This indicates that the bimetallic catalyst exhibits greater catalytic activity than the monometallic catalysts. Comparison with previous studies also supports the excellent catalytic efficiency of Ag–Cu BMNP catalyst based on the high $k_{\mathrm{app}}$ value (**Table S2**).


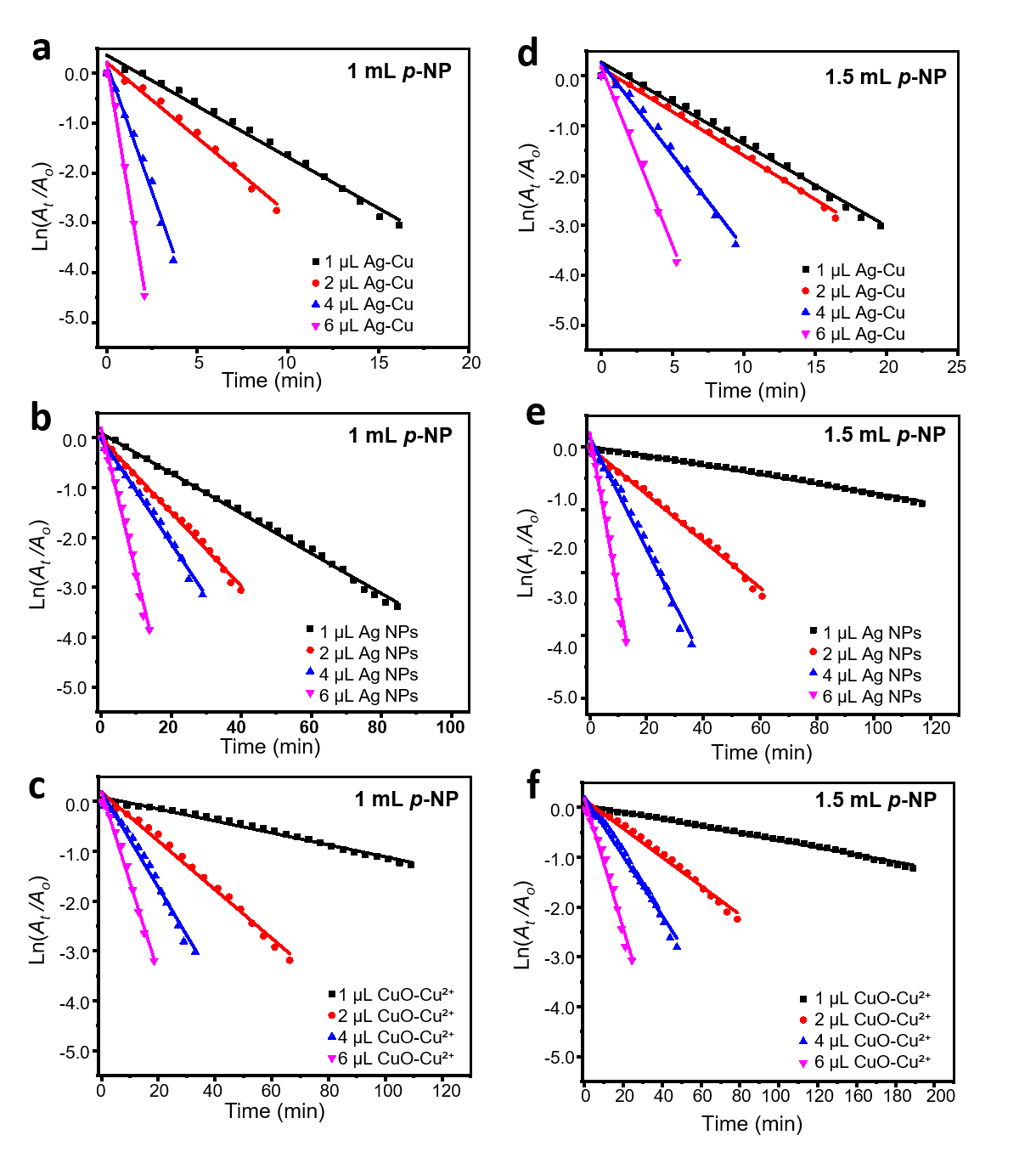


**Figure S11.** (a–c) Kinetic conversion plot of ln(A_t_/A_o_) versus time of the catalyzed conversion of 0.1-mM *p*-NP to *p*-AP by Ag–Cu BMNPs, Ag NPs, and CuO–Cu²⁺.(d–f) Kinetic conversion plot of ln(A_t_/A_o_) versus time of the catalyzed conversion of 0.15-mM *p*-NP to *p*-AP via by Ag–Cu BMNPs, Ag NPs, and CuO–Cu²⁺.

**Table S1**. Kinetic rate constants (*k*_app_) of the synthesized nanocomposites regarding *p-*NP conversion.

| **Catalyst** | **Dose (μL)** | **Rate constant, *k*_app_ (min^−1^)** | |
| --- | --- | --- | --- |
|  |  | **1.5 mL** | **1 mL** |
| **Ag–Cu BMNPs** | 1 | 0.1725 | 0.2071 |
|  | 2 | 0.1831 | 0.3225 |
|  | 4 | 0.3855 | 1.065 |
|  | 6 | 0.7595 | 2.28 |
|  |  |  |  |
| **Ag NPs** | 1 | 0.0076 | 0.0412 |
|  | 2 | 0.0377 | 0.077 |
|  | 4 | 0.092 | 0.1113 |
|  | 6 | 0.2676 | 0.3045 |
|  |  |  |  |
| **CuO–Cu²⁺** | 1 | 0.0071 | 0.012 |
|  | 2 | 0.0298 | 0.0498 |
|  | 4 | 0.0612 | 0.099 |
|  | 6 | 0.1355 | 0.1902 |

**Table S2.** Comparison with literature values.

| **Catalyst structure** | **Pollutant *p*-NP, concentration** | **Rate constant (K_app_), (min^−1^)** | **Reference** |
| --- | --- | --- | --- |
| Ag-Cu | 0.1 mM | 2.28 | Our work |
| Ag-Cu | 0.15 mM | 0.759 | Our work |
| HPAPH/AgNPs | 0.2 mM | 0.358 | [2] |
| Ag@micelle | 0.05 | 1.2 | [3] |
| Ag/Nanosilica | 0.1 | 0.0459 | [4] |
| copper-cotton cellulose (Cu-CC) | 0.13 mM | 9.94 × 10^−3^ | [5] |
| Cu/CNTs nanocomposites | 0.12 | 0.0532 | [6] |
| Fe_3_O_4_@AMALG8@Ag | 0.05 mM | 0.449 | [7] |
| PP-g-EDA@Ag/Cu (M_1_) | 1 | 0.2480 | [8] |
| Ag-Cu (S1) | 0.059 | 0.178 | [9] |
| Ag-Cu (S2) | 0.059 | 0.053 | [9] |
| PET2-APTES-Ag/Cu | 0.033 mM | 0.177 | [10] |

1. **Reusability Test for PETE@Ag–Cu and ACF@Ag–Cu Catalytic Membranes**


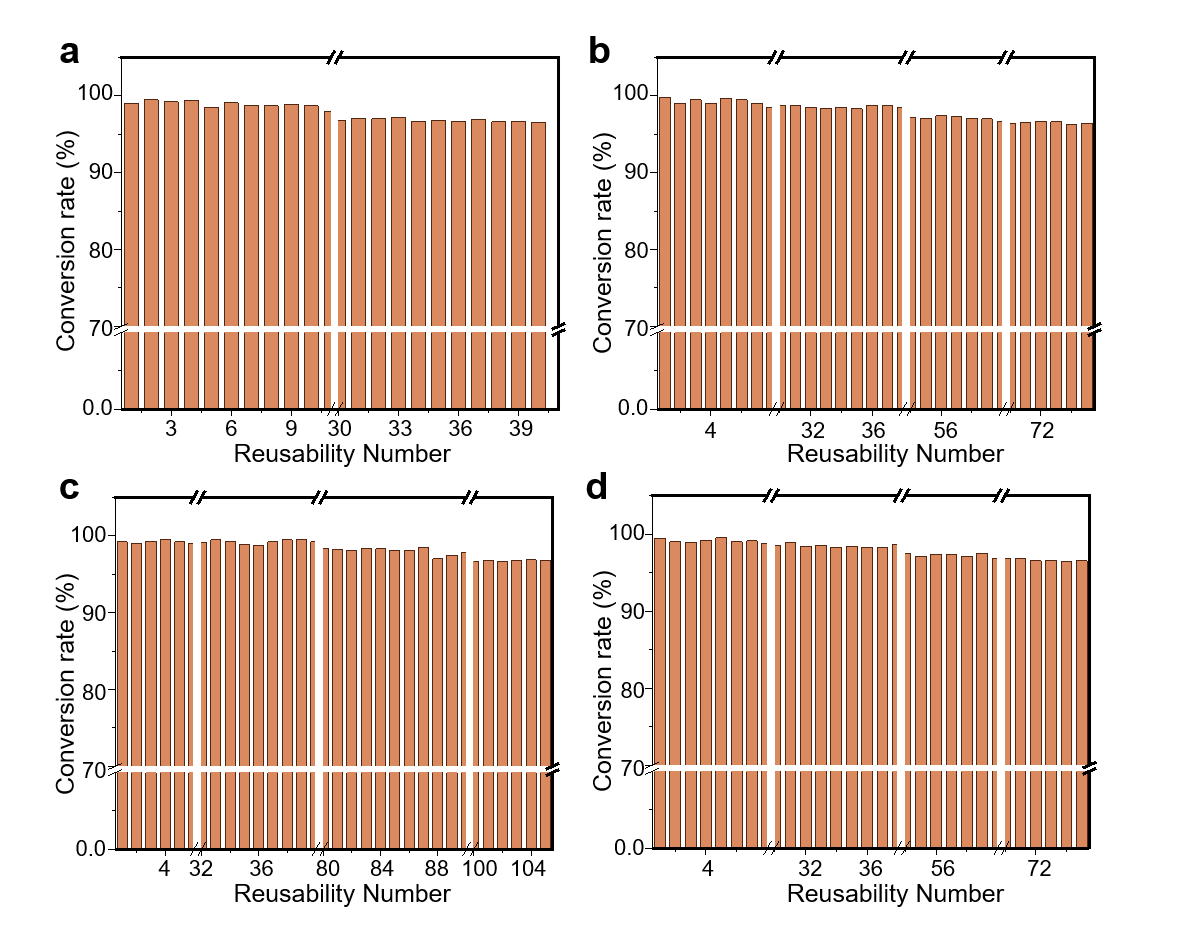


**Figure S12.** (a–c) Reusability of PTFE@Ag–Cu catalytic membrane for complete conversion of 0.15-mM of *p-*NP to *p*-AP at a pumping rate of 20 mL/min for Ag–Cu in (a) 0.25 mL, (b) 0.5 mL, and (c) 1 mL. (d) Reusability of ACF@Ag–Cu catalytic membrane for complete conversion of 0.15-mM of *p-*NP to *p*-AP at a pumping rate of 10 mL/min.


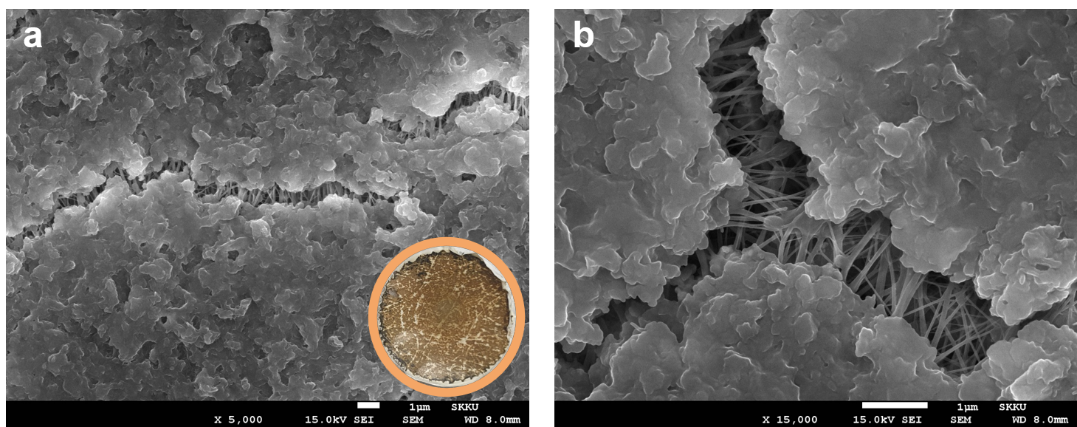


**Figure S13.** (a, b) SEM images of Whatman filter membrane immobilized with Ag–Cu BMNPs (PTFE@Ag–Cu) with different magnifications after 105 cycles of usage at a pumping rate of 20 mL/min (the inset is a picture taken after 105 cycles of usage).

1. **Toxicity Test for Ag NPs, CuO–Cu²⁺, and Ag–Cu BMNPs**

The toxicity was determined using an MTT test protocol according to ISO 10993-5:2009 [2] on fibroblast cells (L-929). First, the L-929 cells were grown in 100-cm^2^ tissue culture flasks in MEM containing 10% FBS and 1% penicillin. Second, the L-929 cells were maintained in a humidified environment of 5% CO_2_ and 95% air at 37°C. After 3 days of incubation, the L-929 cells were harvested via 0.25% trypsin and then centrifuged at 1,500 rpm for 5 min. Next, they were diluted into 1 × 10^5^ cells per 100 μL in each well of 96-well plates and incubated for 24 h. For testing the toxicity of the samples, the previous culture medium (after 24 h) was discarded and replaced with 100 μL of each sample at different doses in the MEM solution for another 24 h in the incubator. MEM (100 μL) was used as a blank sample. The cytotoxicity was evaluated *in vitro* using MTT dye based on mitochondrial dehydrogenase, in which MEM was discarded from each well and replaced with 50 μL of the MTT dye solution. Then, 50-μL MEM was added, and the resulting mixture was kept for an additional 4 h in an incubator at 37°C. Finally, the solution in each well was discarded, and 100-μL DMSO was used to solubilize the reduced formazan crystals before recording the optical density using a microplate reader at a 570-nm wavelength. All experiments were repeated thrice, and the results were averaged.


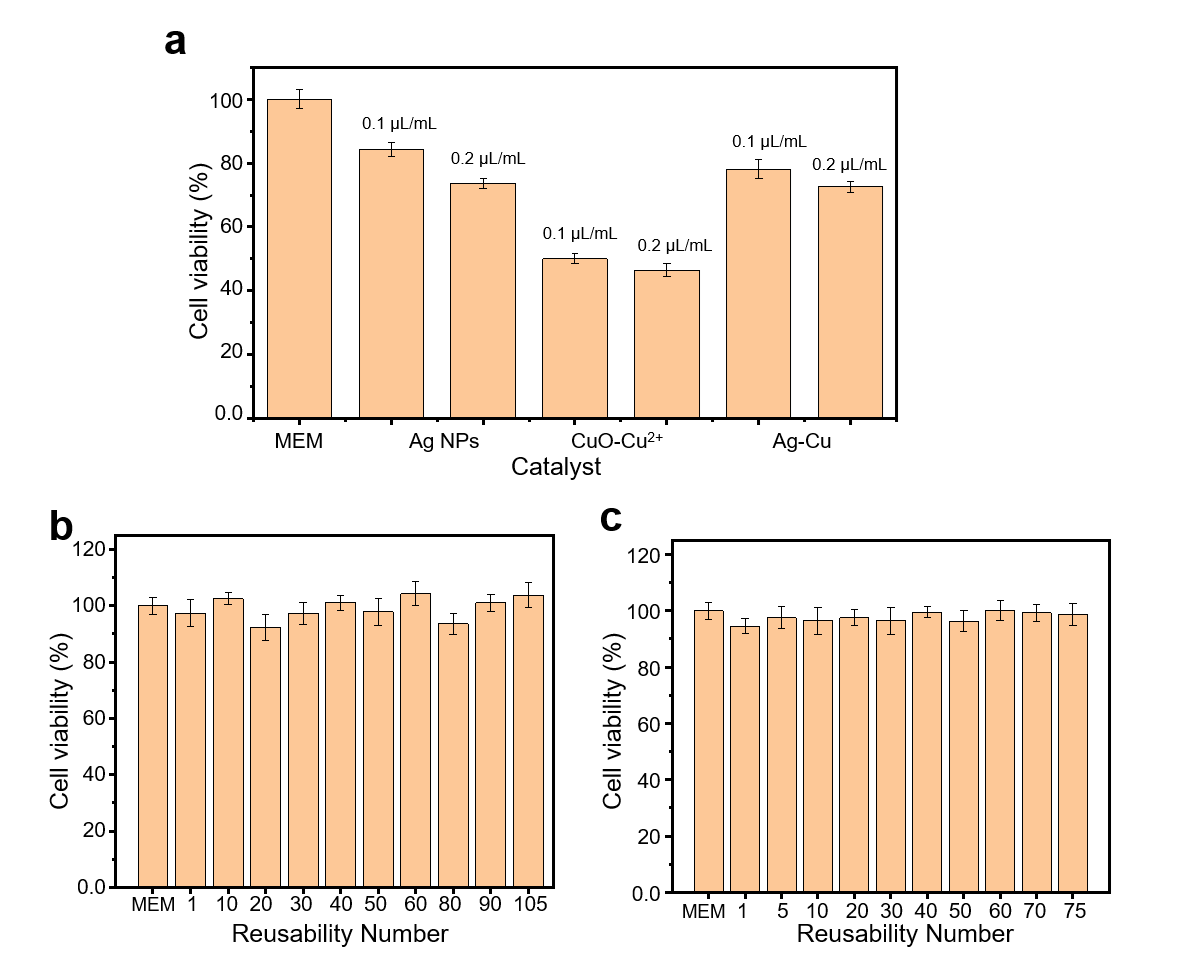


**Figure S14.** (a) Cell viability of synthesized catalysts (Ag NPs, Ag–Cu BMNPs, and CuO/Cu²⁺) at different concentrations higher than those used in catalytic activity; (b) cell viability of the drain water from PTFE@Ag–Cu; (c) cell viability of the drain water from ACF@Ag–Cu. (mean ± SD, n = 3).

1. **Comparison of UV-vis/Time Data for OPD Conversion to DAP Using Ag–Cu BMNPs, Ag NPs, and CuO–Cu²⁺**


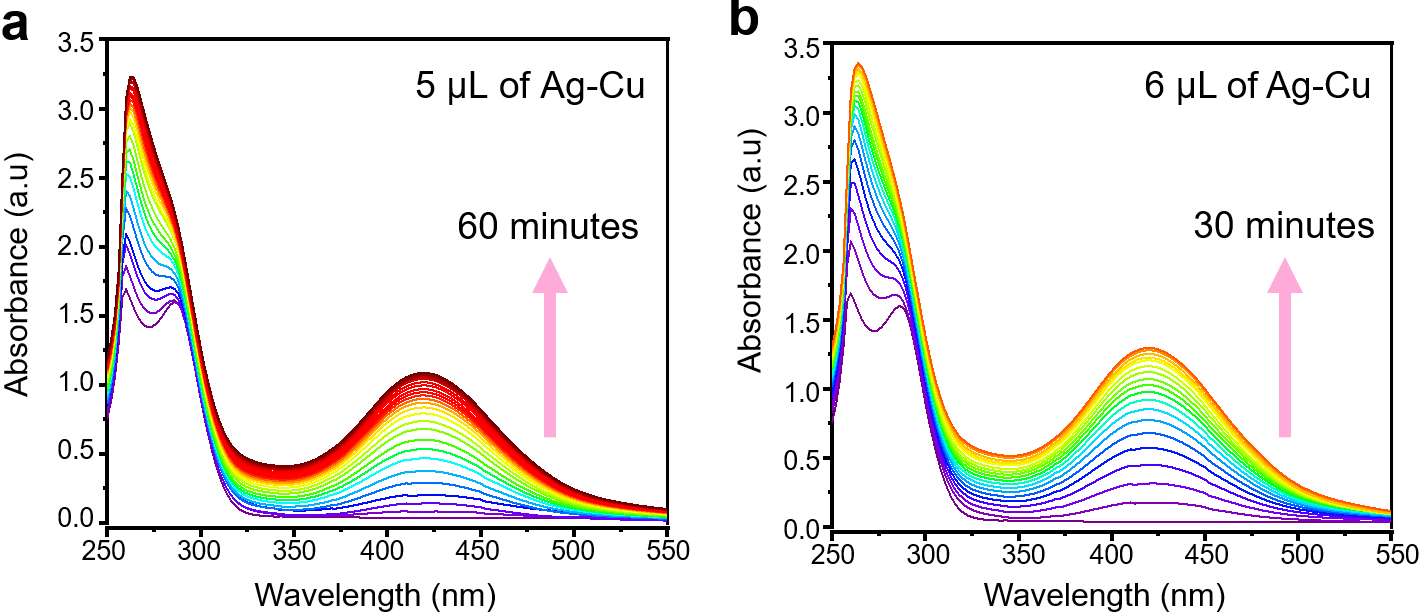


**Figure S15.** UV-vis/time regarding OPD conversion to DAP via Ag–Cu bimetallic nanoenzyme: (a) 5 μL and (b) 6 μL per 50-mL H_2_O_2_ and OPD aqueous solution with final concentrations of 100 and 0.5 mM, respectively.


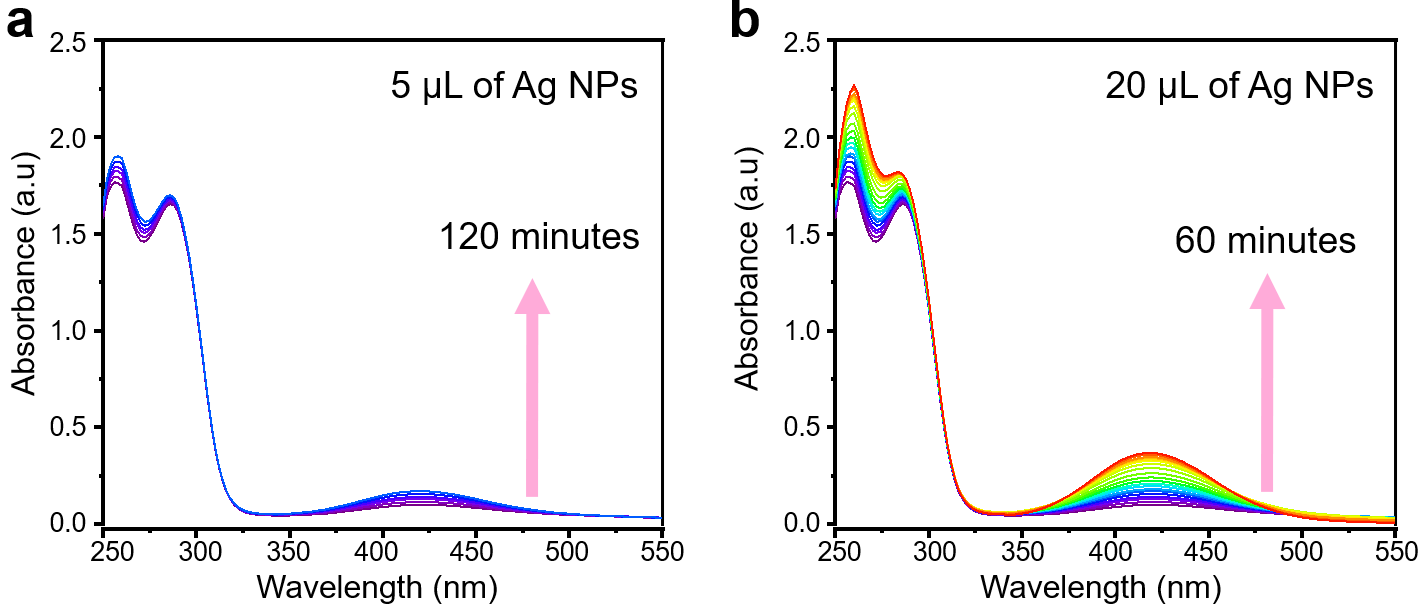


**Figure S16.** UV-vis/time regarding OPD conversion to DAP via Ag NPs: (a) 5 μL and (b) 20 μL per 50-mL H_2_O_2_ and OPD aqueous solution with final concentrations of 100 and 0.5 mM, respectively.


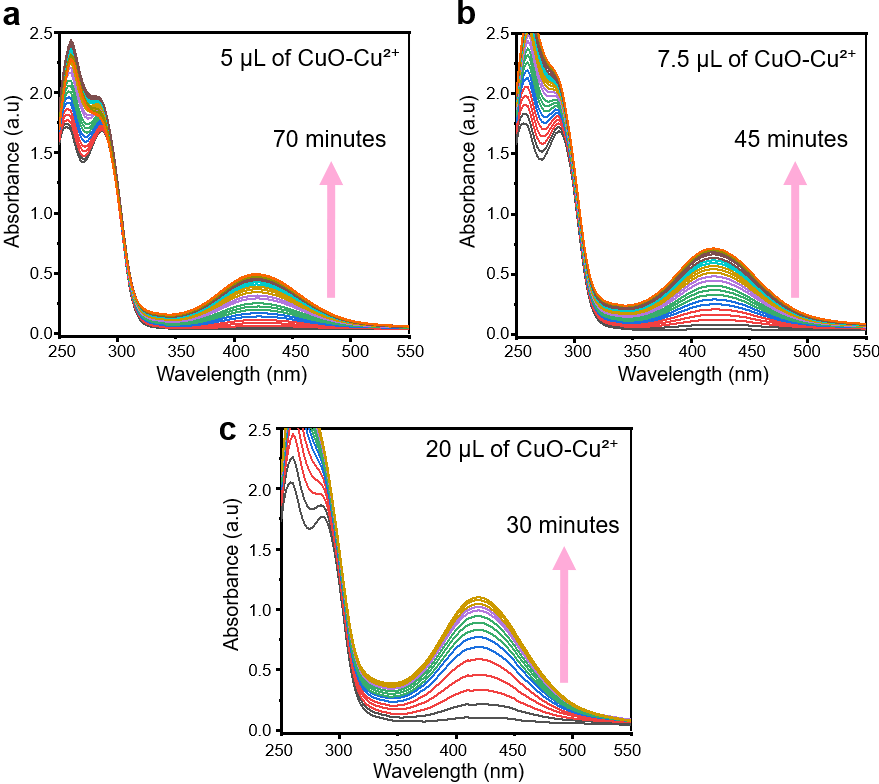


**Figure S17.** UV-vis/time regarding OPD conversion to DAP via CuO–Cu²⁺: (a) 5 μL, (b) 7.5 μL, and (c) 20 μL per 50-mL H_2_O_2_ and OPD aqueous solutions with final concentrations of 100 and 0.5 mM, respectively.

1. **Detailed** **Mechanism for Peroxidase Activity**

The proposed mechanism for the peroxidase-mimic activity mainly depends on the reactive OH• generation and electron transfer processes [63]. First, OPD and H_2_O_2_ can be adsorbed on the surface of the synthesized catalyst, which is facilitated by the presence of vast functional groups of the CTAB/GPEOL surfactant mixture that coats the catalyst, as confirmed by SEM-EDX. The surfactant mixture provided different kinds of interactions, such as hydrogen bonding, electrostatic interaction, and coordination [64]. H_2_O_2_ can be decomposed to reactive OH• by the catalyst action, which is highly promoted in the case of Cu-based catalysts (Ag–Cu and CuO–Cu²⁺) due to the Fenton-like reaction Cu²⁺→Cu¹⁺[64]. Then, OPD can smoothly diffuse into the catalyst surface through various kinds of interactions, facilitating its oxidation into orange-yellow DAP by the action of the generated OH•. Therefore, the Ag–Cu bimetallic catalyst exhibited higher peroxidase-mimic activity than CuO–Cu²⁺ and Ag NPs. This may be due to (i) the synergistic effect between Ag and Cu improving catalytic activity [65], (ii) the higher ability of the Cu-based catalysts (Ag–Cu and CuO–Cu²⁺) to generate OH• via Fenton-like reaction, and (iii) the higher conductivity Ag–Cu BMNPs over single-layer catalysts, acting as an electronic relay for transferring electronics to the adsorbent, thereby facilitating the oxidation process.

1. **Preparation Method of Modified GCE Using Ag–Cu BMNPs**

GCE was thoroughly cleaned using alumina powder, followed by sonication in an ethanol–water mixture to remove any adsorbents from the surface. The synthesized Ag–Cu bimetallic catalyst was centrifuged at 10,000 rpm for 10 min to remove unreacted surfactants. The resulting Ag–Cu powder was redispersed in water, and a 7-µL aliquot was drop-cast onto the GCE, which was then allowed to be dried at 30°C. This process was repeated four times to achieve a uniform catalyst layer, which could provide maximum catalytic activity toward the analytes. Next, 7 µL of 1% Nafion in ethanol was drop-cast over the Ag–Cu layer to strongly bind the catalyst to the GCE. The GCE was dried at room temperature and labeled as GCE@Ag–Cu. For comparison, Ag NPs were also applied to a GCE, labeled as GCE@Ag.

1. **H_2_O_2_ and Glucose Detection Using GCE@Ag–Cu**

GCEs were used to detect glucose and H_2_O_2_. The electrochemical behavior of GCE@Ag and GCE@Ag–Cu in PBS is shown in **Figure S18a**. GCE@Ag exhibited two dissolution peaks at 0.098 and −0.96 V for the oxidation of Ag(0) and their corresponding reduction peak (**Figure S18a**: Curve 1). For GCE@Ag–Cu, the presence of Cu NPs was confirmed from the dissolution peak observed at −0.08 V and their reduction at −0.16 V, along with the redox behavior of Ag NPs (**Figure S18a**: Curve 2). Curves 1 and 2 confirm the successful immobilization of Ag NPs and Ag–Cu BMNPs on the GCE surface, respectively. **Figure S18b** shows the electrocatalytic activity of bare GCE, GCE@Ag, and GCE@Ag–Cu toward H_2_O_2_. There was no change in the electrochemical response of bare GCE before (Curve 1) and after the addition of 1-mM H_2_O_2_ (Curve 2). Meanwhile, the reduction peaks were observed at −0.52 V (Curve 3) and −0.42 V (Curve 4) for GCE@Ag and GCE@Ag–Cu, respectively, after the 1-mM H_2_O_2_ addition. The integration of Cu with Ag in the Ag–Cu bimetallic structure shifted the reduction potential by 100 mV, with a significant shift in the onset potential from −0.3 to 0 V, and increased the current 1.7 times.

The kinetics behind the H_2_O_2_ reduction on GCE@Ag–Cu was further monitored by recording the CVs at different scan rates (100–700 mVs^−1^; **Figure S18c**). The reduction peak current of H_2_O_2_ increased gradually with increasing scan rate, exhibiting a significant linear relationship between the scan rate and current (R^2^ = 0.989). These results suggested that the electrochemical H_2_O_2_ reduction at Ag–Cu followed a diffusion-controlled process [11]. The mechanistic pathway of H_2_O_2_ reduction followed the well-known disproportionation of H_2_O_2_ to oxygen and water on the silver surface, which was enhanced by the presence of Cu° via synergistic effect and improved electrocatalytic activity [12-14].

An amperometric *I*–*t* curve was used to achieve sensitive detection of H_2_O_2_ in PBS (7.4), using GCE@Ag–Cu at concentrations of 1 µM to 2.5 mM, with continuous stirring at a constant potential of −0.8 V. The reduction current increased upon adding 1-µM H_2_O_2_ (**inset**) and further increased with each 50-s interval as the H_2_O_2_ concentration increased (**Figure S18d**). H_2_O_2_ concentration was plotted against the current response, resulting in a significant linear relationship (R^2^ = 0.989; **Figure S18d**, inset). The LOD was 63 nM at a sensitivity of 4,523 µA µM^−1^ cm^−2^. To test the practical applicability, selective detection of H_2_O_2_ in the presence of possible biological interference was performed. The current response for 500-µM H_2_O_2_ did not vary after the injection of 10-mM (1) UA, (2) DA, (3) L-AA, (4) urea, and (5) NaCl (**Figure S18e**). Comparing the recorded results with previous Cu-based electrochemical H_2_O_2_ sensors (**Table S3**), it is evident that our sensor is applicable for the sensitive and selective detection of H_2_O_2_ in biological samples.

The electrochemical behavior of Ag NPs-, CuO–Cu²⁺, and Ag–Cu-modified GCEs was studied by measuring the charge transfer resistance (R_CT_) in the electrode–electrolyte interface using electronic impedance spectroscopy. **Figure S18f** shows Nyquist plots for the modified GCEs in 1-mM [Fe(CN)_6_]^3−/4−^-containing PBS, where the semicircle parts provide information on *R_CT_*. The *R_CT_* values of bare GCE (Curve 1), GCE@CuO-Cu²⁺ (Curve 2), GCE@Ag (Curve 3), and GCE@Ag–Cu (Curve 4) were 30, 14, 6.8, and 1.2 kΩ, respectively. This demonstrates an increase in the electronic conductivity of the GCE after modification with catalysts. Further, the heterogeneous rate constant for electron transfer ($K_{et}$) was calculated as follows:

$K_{et}=\frac{RT}{{n^{2}F^{2}AC^{\circ}R}_{CT}}$^,^  (S5)

where C^0^ denotes the concentration of the [Fe(CN)_6_]^3−/4−^ solution, *A* denotes the electrode surface area (1 cm^2^), n is the number of electrons involved, and *R*, *T*, and *F* are standard usual constants [15]. The $K_{et}$ values were 1.6 × 10^−4^, 7.6 × 10^−3^, 1.19 × 10^−3^, and 1.02 × 10^−3^ cm s^−1^ for bare GCE, GCE@CuO–Cu²⁺, GCE@Ag, and GCE@Ag–Cu, respectively, indicating that the electron transfer reaction was fastest for GCE@Ag–Cu.


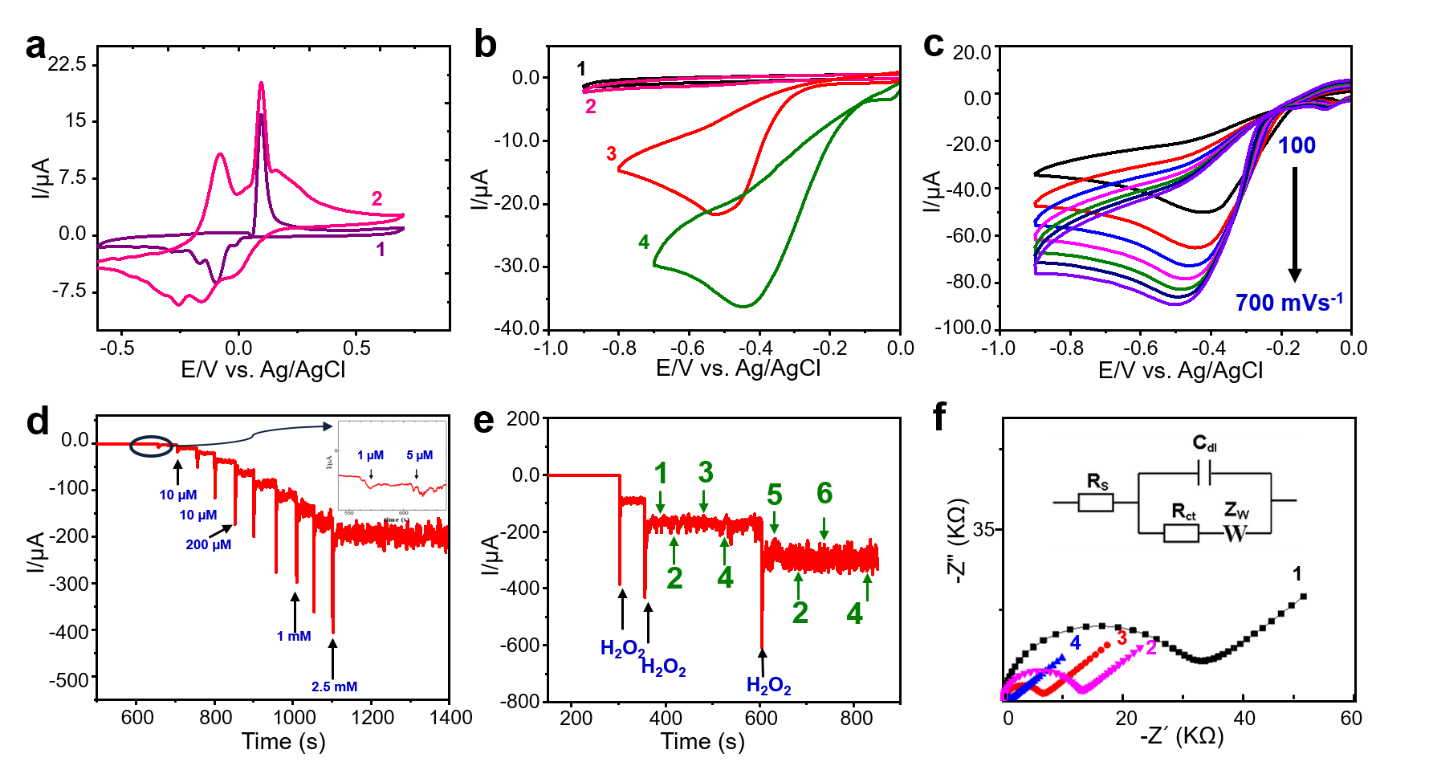


**Figure S18**. Electrochemical characterization of GCEs modified with Ag NPs, and Ag–Cu BMNPs: (a) CVs of GCE@Ag (Curve 1) and GCE@Ag–Cu (Curve 2) in PBS (pH 7.4) at a scan rate of 50 mVs^−1^; (b) CVs of GCE@Ag (Curve 3) and GCE@Ag–Cu (Curve 4) for 1-mM H_2_O_2_ in PBS at a scan rate of 50 mVs^−1^; (c) CVs of GCE@Ag–Cu for 1-mM H_2_O_2_ at varying scan rates (100−700 mVs^−1^); (d) amperometric response of GCE@Ag–Cu for different concentrations of H_2_O_2_ (1 µM–2.5 mM) in PBS (inset: calibration plot of H_2_O_2_ concentration vs current); (e) selective determination of H_2_O_2_ at GCE@Ag-Cu in the presence of interferents (1) UA, (2) DA, (3) AA, and (4) urea; (f) Nyquist plots of (1) bare GCE, (2) GCE@CuO–Cu²⁺, (3) GCE@Ag, and (4) GCE@Ag–Cu in 1 mM [Fe(CN)_6_]^3−/4−^-containing PBS at varying scanning frequencies (0.01 to 100000 Hz; Inset: equivalent circuit).

**Table S3.** Comparison of the prepared H_2_O_2_ sensor with previous Cu-based bimetallic catalysts.

| **No.** | **Catalyst** | **Medium** | **LOD** (µM) | **Ref.** |
| --- | --- | --- | --- | --- |
| **1** | Ag–Cu/ GCE | pH 7 | 0.063 | Present study |
| **2** | Ag–Cu/GCE | pH 7.4 | 152 | [14] |
| **3** | Au–Cu/GCE | pH 7 | 0.73 | [16] |
| **4** | CuO–Ag/Polyimide film | pH 7.4 | 4 | [17] |
| **5** | RGO/Ag-Au/Cu2O/GCE | pH 7.4 | 0.1 | [18] |
| **6** | Ag/Cu_2_O Cubes | pH 7.4 | 0.7 | [19] |
| **7** | Cu_2_O/GNs/GCE | pH 7 | 20.8 | [20] |
| **8** | CQDs/Cu_2_O/GCE | pH 7 | 2.8 | [21] |
| **9** | Ag/FeS_2_/ITO | pH 6 | 0.6 | [22] |

**Table S4.** Comparison of the prepared sensor with previous Cu-based bimetallic catalysts for nonenzymatic glucose detection.

|  | **Catalyst** | **Linear range**  **(µM)** | **Detection limit**  **(µM)** | **Ref.** |
| --- | --- | --- | --- | --- |
| **1** | Ag–Cu/ GCE | 0.5- 3000 | 0.1 | Present study |
| **2** | Ni–Cu/TiO_2_NTs | 10-3200 | 5 | [23] |
| **3** | CuO/TiO_2_/Ti | Up to 2.0 mM | 1 | [24] |
| **4** | Au-Cu/SPE | 250-10,000 | 1.88 | [25] |
| **5** | Co-Cu/FTO | 5-1000 | 0.4 | [26] |
| **6** | Co-Ni-Cu | 50-1551 | 0.5 | [27] |
| **7** | Ni–Cu ANPs/RGO | 0.01–30 | 0.005 | [28] |
| **8** | Cu–Ni/NF | 1–600 | 2 | [29] |
| **9** | rGO@Co_3_O_4_-NC/ITO | 0.5–20 | 0.0504 | [30] |
| **10** | Ni-Cu/CNT/FTO | 20−4500 | 2 | [31] |
| **11** | Co_3_O_4_/CuO/carbon cloth | 1–500 | 380 | [32] |
| **12** | Cu-Ag | Up to 10 μΜ | 2.8 | [33] |
| **13** | Cu-CoNSs | 150-1210 | 10 | [34] |

1. **Synthesis of Gemini Nonionic Amphiphiles**

Three gemini nonionic amphiphiles based on polyethylene oxide were prepared in three steps. First, esterification between fumaric acid and 3-(4-hydroxyphenyl) propanoic acid is performed to prepare diester Compound 1, where fumaric acid (0.04 M, 4.64 g) and 3-(4-hydroxyphenyl) propanoic acid (0.08 M, 13.28 g) were dissolved in 150-mL xylene containing 0.01-g PTSA as a catalyst, and then the reaction mixture was refluxed in Dean–Stark apparatus for receiving the byproduct water (0.72 mL) [35]. The second step involves esterification between Compound 1 (0.006 M, 2.46 g) and polyethylene glycol 1500 (0.012 M, 18 g) following the same procedures mentioned in the first step to obtain on Compound 2 after receiving 0.44-mL water in the Dean–Stark apparatus. Finally, after drying and recrystallization, Compound 2 (0.003 M, 10 g) was esterified with dodecanoic acid (0.006 M, 1.2 g) in 150-mL xylene containing 0.01-g PTSA as catalyst (**Figure S19**). After reaction completion (receiving 0.22 mL H_2_O in Dean–Stark apparatus), the product was washed and recrystallization to obtain the final gemini nonionic surfactant labeled as GPEOL (**Figure S19**).

**Figure S19.** Synthetic routes describing the preparation of GPEOL.

The chemical structure of GPEOL was confirmed by FTIR and ^1^H-NMR spectroscopy (**Figures S20–22**). **Figure S20** shows the FTIR spectra of the starting reagents as received without any purifications. **Figure S21** shows the synthesized GPEOL. **Figure S21** confirmed the synthesized ester material formation, as confirmed by the appearance of a strong band at 1733 cm^−1^, which is indexed for –C=O of ester, while both hydroxyl and carbonyl groups related to carboxylic acid disappeared. Some other bands at 3028, 29020 2871, 1450, and 1350 cm^−1^ correspond to aromatic protons, aliphatic asymmetric and symmetric –C–H, CH_2_ bending, and CH_3_ bending, respectively. The band at 1640 cm^−1^ is ascribed to the C=C double bond, while that in the range of 1100–1255 cm^−1^ is ascribed to the C–O ether group of ethylene oxide. Finally, the two bands at 848 and 950 cm^−1^ are attributed to the –C=C– of the para-substituent aromatic ring.

The number and distribution of protons in GPEOL were confirmed by ^1^H-NMR spectra (**Figure S22**). The ^1^H-NMR spectra of GPEOL shows the following signals: δ = 0.85 (t,6H, **2CH_3_** alkyl chain), δ = 1.25 (m,32H, ــــ 2 COCH_2_CH_2_**(CH_2_)_8_**CH_3_), δ = 1.48 (m,4H, 2COCH_2_**CH_2_**(CH_2_)_8_CH_3_), δ = 2.19 (m,4H, 2CO**CH_2_**CH_2_(CH_2_)_8_CH_3_), δ = 2.55 (m,4H, 2 –Ph-CH_2_**CH_2_**COO–), δ = 2.74 (m,4H, 2 –Ph-**CH_2_**CH_2_COO–), δ = 3.37–3.57 (m,272H, poly ethylene oxide group), δ = 4.56 (s,2H, –C**H**=C**H**–), δ = 6.6 (d,4H, meta-aromatic protons), and δ = 7 (d,4H, ortho-aromatic protons).

**Figure S20.** FTIR of fumaric, hydroxyphenyl propanoic acid, and polyethylene oxide

**Figure S21.** FTIR spectra of GPEOL

**Figure S22.** ^1^HNMR spectra of GPEOL.

1. **(Video S1): Fabrication of Ag-Cu bimetallic using individual and mixed surfactant of CTAB and GPEOL.**


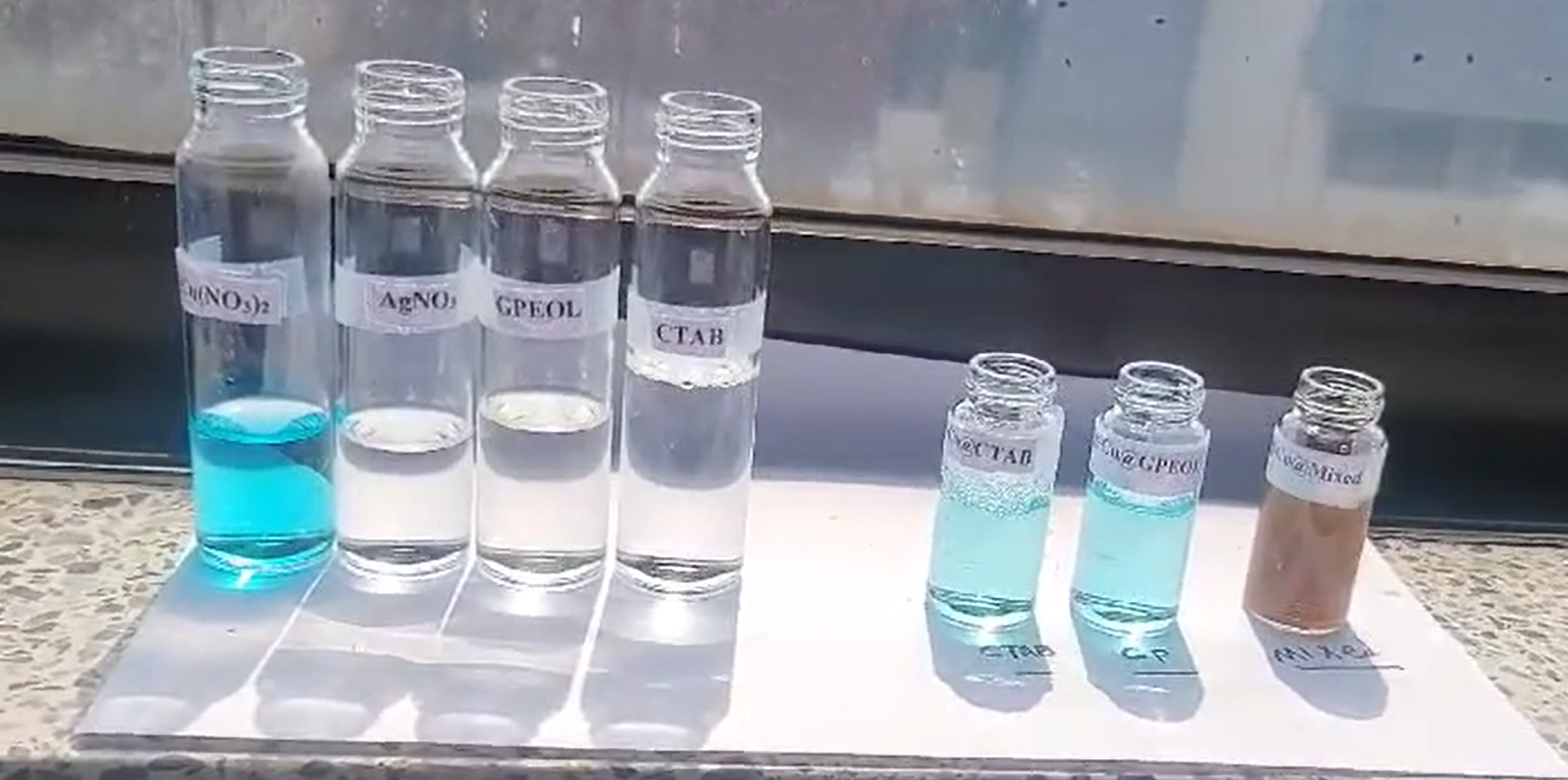


Full video demonstration is available in Video S1.

1. **(Video S2): Performance of PTFE@Ag-Cu Catalytic Membrane in Converting p-NP to p-AP**


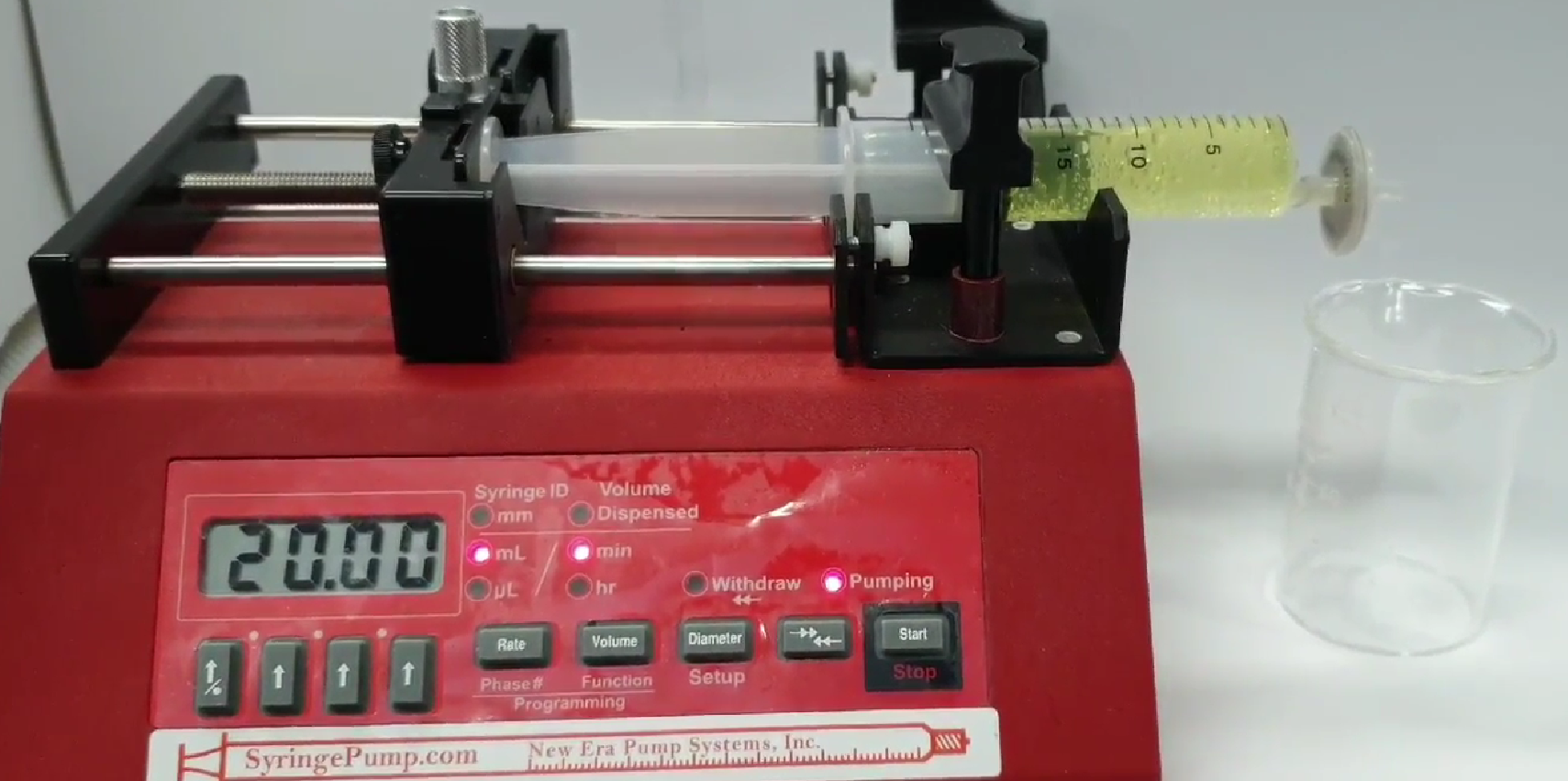


Full video demonstration is available in Video S2.

1. **(Video S3): Performance of ACF@Ag-Cu Catalytic Membrane in Converting p-NP to p-AP**

**
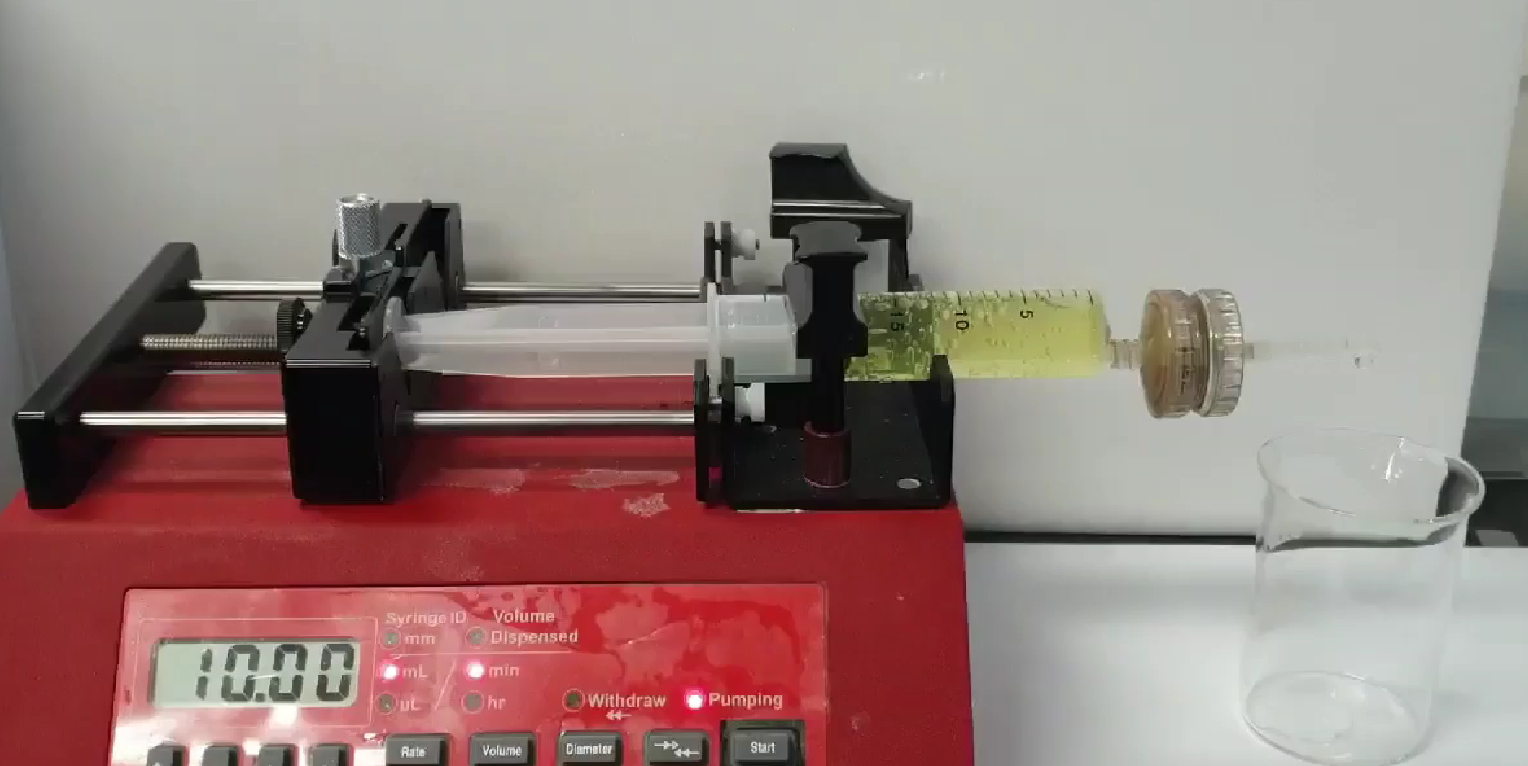
**

Full video demonstration is available in Video S3.

1. **(Video S4): Peroxidase-Mimic Activity of Ag–Cu BMNPs (5 uL).**

**
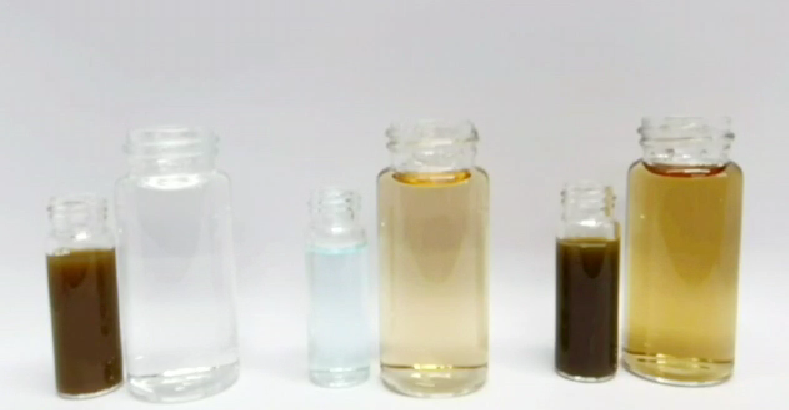
**

Full video demonstration is available in Video S4.

1. **(Video S5): Peroxidase-Mimic Activity of Ag–Cu BMNPs (7.5 uL).**

**
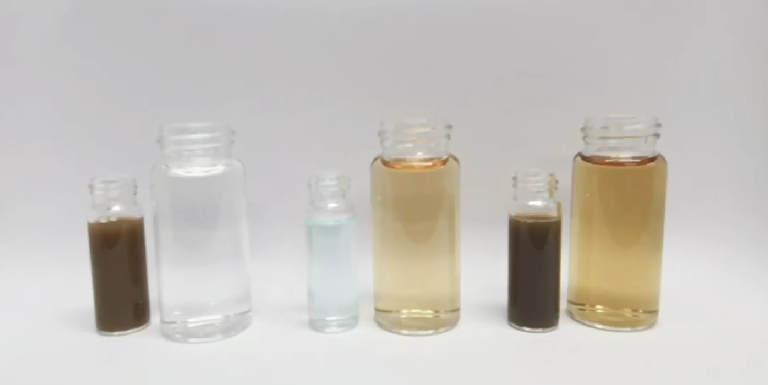
**

Full video demonstration is available in Video S5.

**Supporting References**

1. Badr, E.A., et al., *Synthesis of Schiff base-based cationic Gemini surfactants and evaluation of their effect on in-situ AgNPs preparation: Structure, catalytic, and biological activity study.* MOL. LIQ., 2021. **326**: p. 115342.

2. Elsherif, A., A.H. Elged, and S.M. Shaban, *Controlling effect of hydroxyl phenyl aminopropyl cationic surfactants on the catalytic and biological performance of AgNPs.* Surfaces and Interfaces, 2021. **27**: p. 101530.

3. Geng, Q. and J. Du, *Reduction of 4-nitrophenol catalyzed by silver nanoparticles supported on polymer micelles and vesicles.* RSC Advances, 2014. **4**(32): p. 16425-16428.

4. Das, S.K., et al., *Bio-inspired fabrication of silver nanoparticles on nanostructured silica: characterization and application as a highly efficient hydrogenation catalyst.* Green Chemistry, 2013. **15**(9): p. 2548-2557.

5. Khan, S.B., et al., *Polymer supported metallic nanoparticles as a solid catalyst for the removal of organic pollutants.* Cellulose, 2020. **27**(10): p. 5907-5921.

6. Li, C.x., R.t. Huang, and X.y. Shi, *Biosynthesis of Cu nanoparticles supported on carbon nanotubes and its catalytic performance under different test conditions.* Journal of Chemical Technology & Biotechnology, 2020. **95**(5): p. 1511-1518.

7. Ismail, S.M., et al., *Synthesis of silver decorated magnetic Fe3O4/alginate polymeric surfactant with controllable catalytic activity toward p-NP removal and enzymatic-mimic activity for solid-colorimetric H2O2 detection.* Chemical Engineering Journal, 2023. **453**: p. 139593.

8. Zhang, X.-Q., et al., *Bimetallic Ag-Cu nanoparticles anchored on polypropylene (PP) nonwoven fabrics: Superb catalytic efficiency and stability in 4-nitrophenol reduction.* Chemical Engineering Journal, 2021. **408**: p. 128018.

9. Verma, A.D., et al., *Ag-Cu bimetallic nanocatalysts for p-nitrophenol reduction using a green hydrogen source.* Journal of Environmental Chemical Engineering, 2017. **5**(6): p. 6148-6155.

10. Nabil, B., et al., *Development of new multifunctional filter based nonwovens for organics pollutants reduction and detoxification: High catalytic and antibacterial activities.* Chemical Engineering Journal, 2019. **356**: p. 702-716.

11. Devasenathipathy, R., et al., *Simple electrochemical growth of copper nanoparticles decorated silver nanoleaves for the sensitive determination of hydrogen peroxide in clinical lens cleaning solutions.* Sensors and Actuators B: Chemical, 2017. **252**: p. 862-869.

12. Cai, X., et al., *The mechanism of electrochemical reduction of hydrogen peroxide on silver nanoparticles.* Physical Chemistry Chemical Physics, 2018. **20**(3): p. 1608-1614.

13. Campbell, F.W., et al., *Hydrogen Peroxide Electroreduction at a Silver-Nanoparticle Array: Investigating Nanoparticle Size and Coverage Effects.* The Journal of Physical Chemistry C, 2009. **113**(21): p. 9053-9062.

14. Shafa, M., et al., *Ag-Cu nanoalloys: An electrochemical sensor for H2O2 detection.* Surfaces and Interfaces, 2023. **36**: p. 102616.

15. Gowthaman, N.S.K., et al., *Negative Potential-Induced Growth of Surfactant-Free CuO Nanostructures on an Al–C Substrate: A Dual In-Line Sensor for Biomarkers of Diabetes and Oxidative Stress.* ACS Sustainable Chemistry & Engineering, 2020. **8**(7): p. 2640-2651.

16. Gowthaman, N.S.K., S. Shankar, and S. Abraham John, *Substrate catalyzed formation of Au-Cu bimetallic nanoparticles as electrocatalyst for the reduction of dioxygen and hydrogen peroxide.* Journal of Electroanalytical Chemistry, 2018. **812**: p. 37-44.

17. Hooch Antink, W., et al., *Simple synthesis of CuO/Ag nanocomposite electrode using precursor ink for non-enzymatic electrochemical hydrogen peroxide sensing.* Sensors and Actuators B: Chemical, 2018. **255**: p. 1995-2001.

18. Li, D., et al., *Enhanced non-enzymatic electrochemical sensing of hydrogen peroxide based on Cu2O nanocubes/Ag-Au alloy nanoparticles by incorporation of RGO nanosheets.* Journal of Electroanalytical Chemistry, 2017. **791**: p. 23-28.

19. Qi, C. and J. Zheng, *Novel Nonenzymatic Hydrogen Peroxide Sensor Based on Ag/Cu2O Nanocomposites.* Electroanalysis, 2016. **28**(3): p. 477-483.

20. Liu, M., R. Liu, and W. Chen, *Graphene wrapped Cu2O nanocubes: Non-enzymatic electrochemical sensors for the detection of glucose and hydrogen peroxide with enhanced stability.* Biosensors and Bioelectronics, 2013. **45**: p. 206-212.

21. Li, Y., et al., *Carbon quantum dots/octahedral Cu2O nanocomposites for non-enzymatic glucose and hydrogen peroxide amperometric sensor.* Sensors and Actuators B: Chemical, 2015. **206**: p. 735-743.

22. Li, D., et al., *Fabrication of Ag nanoparticles coupled with ferrous disulfide biocatalyst as a peroxidase mimic for sensitive electrochemical and colorimetric dual-mode biosensing of H2O2.* Food Chemistry, 2022. **393**: p. 133386.

23. Li, X., et al., *Nickel/Copper nanoparticles modified TiO2 nanotubes for non-enzymatic glucose biosensors.* Sensors and Actuators B: Chemical, 2013. **181**: p. 501-508.

24. Luo, S., et al., *A new method for fabricating a CuO/TiO2 nanotube arrays electrode and its application as a sensitive nonenzymatic glucose sensor.* Talanta, 2011. **86**: p. 157-163.

25. Ngamaroonchote, A., et al., *Highly branched gold–copper nanostructures for non-enzymatic specific detection of glucose and hydrogen peroxide.* Microchimica Acta, 2020. **187**(10): p. 559.

26. Pak, M., et al., *Cobalt-copper bimetallic nanostructures prepared by glancing angle deposition for non-enzymatic voltammetric determination of glucose.* Microchimica Acta, 2020. **187**(5): p. 276.

27. Gong, X., et al., *High-Performance Non-enzymatic Glucose Sensors Based on CoNiCu Alloy Nanotubes Arrays Prepared by Electrodeposition.* Frontiers in Materials, 2019. **6**.

28. Xu, X., et al., *Non-enzymatic electrochemical detection of glucose using Ni–Cu bimetallic alloy nanoparticles loaded on reduced graphene oxide through a one-step synthesis strategy.* Analytical Methods, 2021. **13**(46): p. 5628-5637.

29. Wei, H., et al., *Dendritic core-shell copper-nickel alloy@metal oxide for efficient non-enzymatic glucose detection.* Sensors and Actuators B: Chemical, 2021. **337**: p. 129687.

30. Zhang, Y., et al., *In situ formation of reduced graphene oxide@Co3O4-N-doped carbon and its structure-function relationship for glucose sensing.* Applied Surface Science, 2021. **539**: p. 148235.

31. Ammara, S., et al., *Nonenzymatic glucose sensor with high performance electrodeposited nickel/copper/carbon nanotubes nanocomposite electrode.* Journal of Physics and Chemistry of Solids, 2018. **120**: p. 12-19.

32. Cheng, S., et al., *Hierarchical Co3O4/CuO nanorod array supported on carbon cloth for highly sensitive non-enzymatic glucose biosensing.* Sensors and Actuators B: Chemical, 2019. **298**: p. 126860.

33. Smikhovskaia, A.V., et al., *In situ laser-induced synthesis of copper‑silver microcomposite for enzyme-free d-glucose and l-alanine sensing.* Applied Surface Science, 2019. **488**: p. 531-536.

34. Wang, L., et al., *Dendritic copper-cobalt nanostructures/reduced graphene oxide-chitosan modified glassy carbon electrode for glucose sensing.* Sensors and Actuators B: Chemical, 2014. **195**: p. 1-7.

35. Shaban, S.M., et al., *Some alginates polymeric cationic surfactants; surface study and their evaluation as biocide and corrosion inhibitors.* J. Mol. Liq., 2019. **273**: p. 164-176.
